# Characterization of HMGB1/2 Interactome in Prostate Cancer by Yeast Two Hybrid Approach: Potential Pathobiological Implications

**DOI:** 10.3390/cancers11111729

**Published:** 2019-11-05

**Authors:** Aida Barreiro-Alonso, María Cámara-Quílez, Martín Salamini-Montemurri, Mónica Lamas-Maceiras, Ángel Vizoso-Vázquez, Esther Rodríguez-Belmonte, María Quindós-Varela, Olaia Martínez-Iglesias, Angélica Figueroa, María-Esperanza Cerdán

**Affiliations:** 1 EXPRELA Group, Centro de Investigacións Científicas Avanzadas (CICA), Departamento de Bioloxía, Facultade de Ciencias, INIBIC-Universidade da Coruña, Campus de A Coruña, 15071 A Coruña, Spainmonica.lamas@udc.es (M.L.-M.);; 2 Translational Cancer Research Group, Instituto de Investigación Biomédica de A Coruña (INIBIC), Carretera del Pasaje s/n, 15006 A Coruña, Spain; 3 Epithelial Plasticity and Metastasis Group, Instituto de Investigación Biomédica de A Coruña (INIBIC), Complexo Hospitalario Universitario de A Coruña (CHUAC), Sergas, 15006 A Coruña, Spain

**Keywords:** two hybrid, interactome, prostate cancer, biomarkers

## Abstract

High mobility group box B (HMGB) proteins are pivotal in the development of cancer. Although the proteomics of prostate cancer (PCa) cells has been reported, the involvement of HMGB proteins and their interactome in PCa is an unexplored field of considerable interest. We describe herein the results of the first HMGB1/HMGB2 interactome approach to PCa. Libraries constructed from the PCa cell line, PC-3, and from patients’ PCa primary tumor have been screened by the yeast 2-hybrid approach (Y2H) using HMGB1 and HMGB2 baits. Functional significance of this PCa HMGB interactome has been validated through expression and prognosis data available on public databases. Copy number alterations (CNA) affecting these newly described HMGB interactome components are more frequent in the most aggressive forms of PCa: those of neuroendocrine origin or castration-resistant PCa. Concordantly, adenocarcinoma PCa samples showing CNA in these genes are also associated with the worse prognosis. These findings open the way to their potential use as discriminatory biomarkers between high and low risk patients. Gene expression of a selected set of these interactome components has been analyzed by qPCR after HMGB1 and HMGB2 silencing. The data show that HMGB1 and HMGB2 control the expression of several of their interactome partners, which might contribute to the orchestrated action of these proteins in PCa

## 1. Introduction

Human high mobility group box B (HMGB) proteins HMGB1, 2, and 3 are differentially expressed in many different tissues and cell types, whereas HMGB4 expression is restricted to the testis [1]. HMGB2 has 82.3% sequence similarity with HMGB1, and both proteins have common or redundant functions in inflammation [2], chromosome remodeling activity [3], V (D) J recombination [4], and embryonic development [5].

HMGB1 has been related to the onset and progression of cancer, being involved in events such as replenishing telomeric DNA and maintaining cell immortality [6], autophagic increase, evasion of apoptosis [7,8], as well as cell proliferation and invasion [9,10]. HMGB1 is also involved in dedifferentiation during epithelial to mesenchymal transition (EMT) [11] via the receptor for advanced glycation endproducts RAGE/ nuclear factor kappaB NF-κB signaling pathways [12] and in angiogenesis [13]. The role of HMGB2 in these processes, although less well studied, has also been related to cell viability and invasion [14], EMT [10], and angiogenesis [15].

The majority of the prostate cancers (PCa) are adenocarcinomas characterized by glandular formation and the expression of androgen receptor (AR) and prostate-specific antigen (PSA). Hormonal inhibition of AR signaling is the therapeutic choice for patients with adenocarcinomas, but unfortunately, the disease usually progresses as it becomes independent of exogenous AR induction, leading to castration-resistant prostate cancer (CRPC) with a worse prognosis. In prostatic small cell neuroendocrine carcinoma (SCNC), the tumor cells are negative for AR and PSA expression and do not respond to hormonal therapy [16]. Among the most frequently used PCa cell lines, PC-3 characteristics are considered closer to a SCCN PCa model and those of DU145 (ATCC^®^ HTB-81™) or LNCaP (lymph node carcinoma of the prostate) are considered closer to adenocarcinoma models [16]. PC-3 and DU145 are AR-independent, and LNCaP is AR-dependent [16,17]. Interestingly, upregulation of HMGB1 mRNA and protein have been detected in PCa tumors [12,18] and PCa cell lines (including PC-3 and DU145 or LNCaP) compared to the non-transformed immortalized prostate cell line RWPE-1 (prostate epithelial transformed by HPV)) [18]. Silencing of HMGB1 in LNCaP cells inhibits cell growth [19]. HMGB1 expression is notably high in PCa metastasis [12] and is positively correlated with some clinical-pathological parameters, such as Gleason score or preoperative PSA concentration, being associated with a worse prognosis [18].

Proteomic studies in relation to PCa have been reported [20,21,22], with interactome strategies being outstanding in recent developments [23,24,25]. The purpose of our study was to analyze proteins interacting with HMGB1 and HMGB2 by the yeast 2-hybrid approach (Y2H), using HMGB1 and HMGB2 baits. Results from the screening of libraries constructed from the PC-3 line, as a model of metastatic AR-independent PCa, and of libraries obtained from PCa adenocarcinoma primary tumor are presented. Analyses of copy number alterations (CNA) and mRNA levels of detected targets in public PCa databases are discussed showing that dysregulation of some HMGB1/2 targets is associated with clinical prognosis. Considering that HMGB proteins are known regulators of gene expression, we also tested whether HMGB1 and HMGB2 silencing affects the expression of their Y2H detected partners and found that this regulatory mechanism is functional in PC-3 cells.

## 2. Results

### 2.1. HMGB1 and HMGB2 Y2H Interactomes in the PCa PC-3 Cell Line and in Adenocarcinoma Primary Tumor

Human PCa cDNA libraries were constructed using total RNA from PC-3 cells and PCa adenocarcinoma primary tumor. Y2H assays were carried out as described in the Materials and Methods section, using HMGB1 and HMGB2 as baits and triple screening by 3 independent selection markers (Supplementary Figure S1). The panel of proteins interacting with HMGB1 or HMGB2 in these libraries is summarized in Table 1, Table 2, Table 3 and Table 4. The interactions of identified proteins with HMGB1 or HMGB2 have not previously been reported on Biogrid, String, or other public databases, although we have previously reported that Cytokeratin-7, the human complement subcomponent C1q (C1QPB), and zinc finger p rotein 428 (ZNF428) interact with HMGB1 and that (high density lipoprotein-binding protein (HDLBP) and ZNF428 interact with HMGB2 in noncancerous epithelial cells [26].

**Figure 1:**
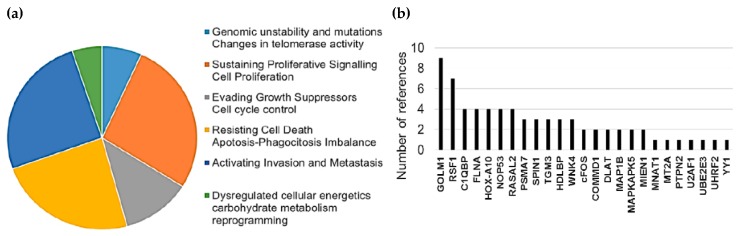
Relationship between identified proteins and cancer hallmarks: (**a**) Distribution of HMGB1 and HMGB2 interactome targets according to cancer hallmarks and (**b**) number of references that associate these proteins with cancer hallmarks according to PubMed (7-31-2019).

Interestingly, 43% of detected HMGB1 interactome targets (10 of a total of 28, referenced in Table 1 and Table 3) as well as 64% of HMGB2 (7 of a total of 11, referenced in Table 2 and Table 4) have previously been related to PCa, supporting the functional significance of our Y2H interactome data in PCa research. Furthermore, the detected proteins are remarkably associated with cancer hallmarks. Indeed, the oncogenic capacities of several identified proteins in our Y2H interactome had been already reported in PCa or other cancerous models by wide-ranging functional approaches, which are reviewed in Supplementary Table S1. Figure 1 summarizes the frequency distribution of the identified proteins in relation to cancer hallmarks (Figure 1a) as well as the number of references of each protein functionally related to cancer progression in diverse models (Figure 1b).

**Figure 2:**
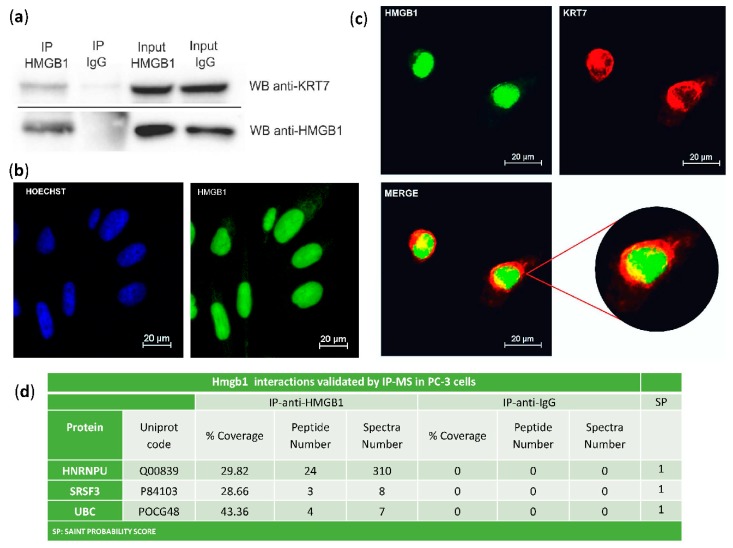
Validation of HMGB1-interactions: (**a**) Cytokeratin-7 co-immunoprecipitation with HMGB1. PC-3 lysates were immunoprecipitated with anti-HMGB1 antibody or normal mouse IgG and immunoblotted with antibodies to Cytokeratin-7 and HMGB1; complete membranes provided as Supplementary Materials Imagen S1. Protein G horseradish peroxidase (HRP)-labelled was used as a secondary antibody to minimize the signal given by the light and heavy chains of the immunoprecipitation antibody. (**b**) Immunofluorescent localization of HMGB1 in PC-3 cells and comparison to Hoechst-stained nuclei. (**c**) Immunofluorescent co-localization of HMGB1 and Cytokeratin-7 by confocal microscopy in PC-3 cells. HMGB1 is shown in green, and Cytokeratin-7 is in red. Co-localization is seen in yellow by merging. (**d**) Validation of interactions with HNRNPU, SRSF3, and UBC after HMGB1 immunoprecipitation and MS peptide identification.

The interaction of HMGB1 with Cytokeratin-7 was validated in PC-3 cells by co-immunoprecipitation and western blot (Figure 2a). Immunodetection of HMGB1 using a green fluorescent antibody (Figure 2b,c) and of Cytokeratin-7 using a red fluorescent antibody (Figure 2c) was also assayed in PC-3 cells. Confocal microscopy showed that co-localization of HMGB1 and Cytokeratin-7 occurred principally in the perinuclear area (Figure 2c), with a Meander’s correlation coefficient of 0.87 ± 0.3. Three other interactions were also validated in PC-3 cells by immunoprecipitation and MS identification (Figure 2d).

### 2.2. Mutations and Copy Number Alterations in HMGB1 and HMGB2 Interactome Targets in PCa

**Figure 3:**
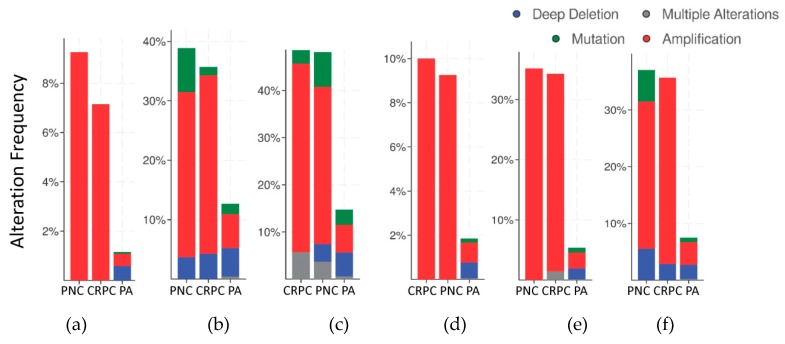
Copy number alteration frequency of HMGB1, HMGB2, and their targets in prostate cancer: (**a**) HMGB1, (**b**) HMGB1 interactome targets from PC-3 library, (**c**) HMGB1 interactome targets from prostate adenocarcinoma tissue library, (**d**) HMGB2, (**e**) HMGB2 interactome targets from PC-3 library, and (**f**) HMGB2 interactome targets from prostate adenocarcinoma tissue library. PA, prostate adenocarcinoma; PNC, Prostate Neuroendocrine Carcinoma; CRPC, Castration Resistant Prostate Cancer. Data source: combined study from data available through c-Bioportal (detailed in Supplementary Table S2).

**Figure 4:**
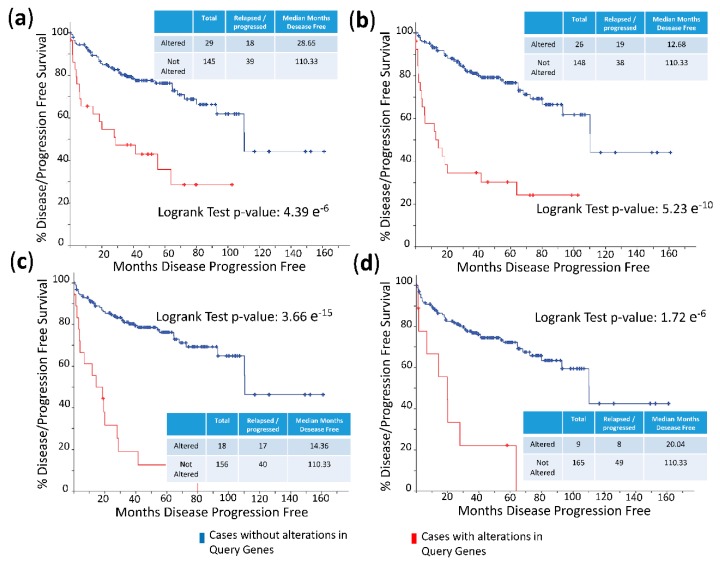
Disease/progression-free Kaplan–Meier estimate: (**a**) Cases altered in HMGB1 interactome targets from PC-3 library; (**b**) cases altered in HMGB1 interactome targets from prostate adenocarcinoma tissue library; (**c**) cases altered in HMGB2 interactome targets from PC-3 library; and (**d**) cases altered in HMGB2 interactome targets from prostate adenocarcinoma tissue library. Data source: prostate adenocarcinoma study [86], including 194 patients/samples.

The frequency of mutations and copy number alterations (CNA) in genes encoding HMGB1 and HMGB2 proteins were analyzed as well as in those genes encoding proteins detected in the Y2H search associated with PCa, using the open platform for exploring cancer genomics data, c-Bioportal [79,80]. We included 14 PCa studies available at cBioPortal (https://www.cbioportal.org/), of which their characteristics are summarized in Supplementary Table S2. From these, 10 were adenocarcinoma studies [81,82,83,84,85,86,87,88,89,90,91], with 3218 samples; the other 3 studies corresponded to metastatic PCa [92,93,94], including 655 samples; and finally, one study corresponded to neuroendocrine PCa, which was carried out with 114 samples [95]. The data show that mutations and CNA affecting HMGB1, HMGB2, and the proteins identified in the corresponding Y2H interactome are more frequently present in neuroendocrine PCa and castration-resistant PCa than in adenocarcinoma (Figure 3). Since neuroendocrine PCa is an aggressive PCa [16], we tested whether CNA of these genes was also related to the poor prognosis in patients diagnosed with adenocarcinoma. With amplification as the most frequently detected CNA in Figure 3, we compared disease/progression-free Kaplan–Meier estimate rates calculated from the study of Taylor et al. [86] among the group of samples having gains or amplifications of these genes and the group integrated by the rest of samples. Figure 4 shows that gain or amplification of HMGB1 and HMGB2 interactome targets results in a notorious decrease of the median of months disease-free, with high significant *p*-values in the Logrank test.

### 2.3. Expression of HMGB1 and HMGB2 Interactome Targets in PCa

**Figure 5:**
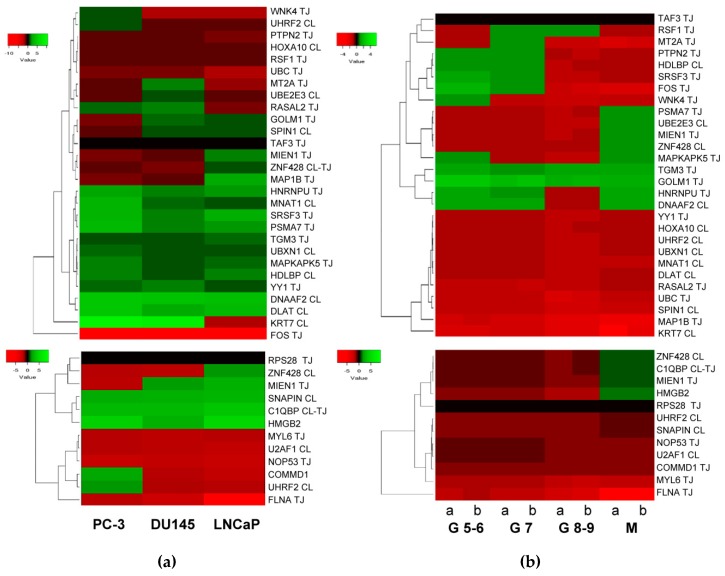
Heat map expression in prostate cancer: (**a**) Expression of HMGB1 interactome targets (upper panel) and HMGB2 interactome targets (lower panel) in 3 prostate cancer cell lines; (**b**) expression of HMGB1 interactome targets (upper panel) and HMGB2 interactome targets (lower panel) in prostate adenocarcinoma cases classified by Gleason (G) score groups or metastatic (M) tumors. CL, target detected in the PC-3 library. TJ, target detected in the prostate adenocarcinoma library. Data extracted from Gene Expression Omnibus (GEO ) Accession: GSE21032.

According to published data [12,18], HMGB1 expression increases in PCa cell lines and tissues from PCa, especially in metastases. With published data of RNA levels in PCa samples [86] retrieved from Geen Expression Omnibus (GEO Accession: GSE21032), the change fold expression of HMGB1 and HMGB2-interactome targets in PCa cell lines versus noncancerous cells was calculated, from which a heat map was constructed (Figure 5a). Using the same source, data was retrieved from 181 adenocarcinoma primary tumors, which were distributed in 3 groups clinically classified by Gleason scores, and in a 4th group integrated by 37 metastatic tumors. The change fold expression of HMGB1 and HMGB2-interactome targets in each group versus noncancerous cells from healthy tissues were calculated, from which the heat map shown in Figure 5b was constructed. The classification of each gene in the main clusters of the heat maps proved to be unrelated to the experimental library origin of the clone (PC-3 cell line or PCa adenocarcinoma primary tumor). The results reveal that genes encoding 11 proteins interacting with HMGB1 (Figure 4a top panel) are also upregulated in the 3 PCa cell lines (PC-3 and DU145 or LNCaP), and 8 more are upregulated in one or two PCa cell lines. Among the detected HMGB2 partners, 2 are upregulated in the 3 PCa cell lines: 1 in 2 and 3 in at least one (Figure 4a, bottom). In both HMGB1 and HMGB2 interactomes, the targets upregulated in metastatic tissue (Figure 5b) are a subset of those upregulated in one or more of the PCa cell lines. Analyzing expression in reference to Gleason score, the genes TMG3 and GOLM1 are upregulated in all the groups, whereas the others are only upregulated in groups classified with a Gleason score of less than or equal to 7 (PTPN2, HDLBP, SRF3, FOS, and WNK4). Regarding a pattern associated with the existence of metastasis, 3 genes that are not upregulated in samples from primary tumors are upregulated in metastasis: PSMA7, UBE2E3, and MIEN1 (Figure 5b).

### 2.4. Silencing of HMGB1 and HMGB2 Reveals Regulation of the Expression of Genes Encoding Their Interactome Targets

**Figure 6:**
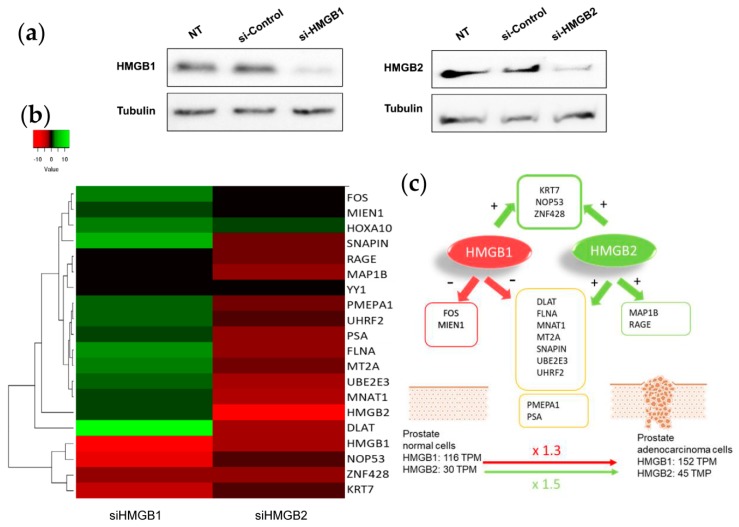
HMGB1 and HMGB2 silencing: (**a**) Western Blot showing HMGB1 and HMGB2 silencing. Complete membranes provided as Supplementary Material, Image S2 (**b**) Heat map comparing the pattern of expression (siHMGB1/HMGB1 and siHMGB2/HMGB2). (**c**) Summary of regulatory effects of HMGB1 and HMGB2 on the selected genes.

To test whether changes in HMGB1/2 protein levels in PCa cells could also be influencing the expression of their interactome targets, HMGB1 and HMGB2 in PC-3 cells were silenced by iRNA (Figure 6a). Levels of mRNA from 14 partners analyzed by qPCR and changes (siHMGB/HMGB) are summarized in the Figure 6b. This analysis also included HMGB1, HMGB2, and well-known PCa biomarkers: PSA (encoded by KLK3); PMEPA1, which is involved in downregulation of the androgen receptor, thus promoting androgen receptor-negative prostate cell proliferation [96]; and RAGE, one of the membrane receptors in the extracellular signaling function of HMGB1 [97]. Silencing of HMGB1 causes overexpression of the larger cluster of the Y2H interactome, whereas siHMGB2 has the opposite effect (Figure 6c). HMGB1 downregulates the expression of the majority of targets analyzed, and conversely, HMGB2 upregulates them. Therefore, the expression level of each regulated target would depend on the relative imbalance of HMGB1 and HMGB2 and on the differential effect of both HMGB proteins on the expression of each partner. PMEPA1 and PSA, well-known PCa biomarkers, are also oppositely regulated by HMGB1 and HMGB2 (Figure 6).

## 3. Discussion

High mobility group box B (HMGB) proteins are pivotal in the development of cancer [6,8,10], and HMGB1 overexpression has been related to principal cancer hallmarks [7]. Interactome targets of HMGB1 or HMGB2 that have been identified in our Y2H study were previously found to be related to cancer hallmarks (Table S1 and Figure 1), and are also dysregulated in PCa, as confirmed by detection of changes in mRNA or protein levels. DNAAF2 [98], U2AF1 [43], C1QBP [40], Snapin, or HDLBP [99] are upregulated in prostate tumors or PCa cell lines. Others increase their expression after androgen-deprivation therapy, such as KRT7 or NOP53 [100]. Functional studies interfering the expression of several of the proteins revealed by our study also directly associated them to PCa. In this sense, selective knockdown of C1QBP through iRNA decreased cyclin D1, increased p21 expression, led to cell cycle arrest (G1/S transition) in PCa cells, and had no effect on a noncancerous cell line [40]. NOP53 acts as a tumor suppressor, and knockdown of the gene in the PCa LNCaP cell line increased the invasiveness of these cells as measured in a xenograft animal model [101].

Two already known regulatory factors have been found among the HMGB1 interactome targets, YY1 and HOXA10, and both are associated with PCa. YY1 is upregulated in human prostate cancer cell lines and tissues [66]. Inhibition of YY1 reduces expression of genes related to the Krebs cycle and electron transport chain in PCa cell lines [67], and YY1 depletion correlates with delayed progression of PCa [68]. Overexpression of YY1 can promote epithelial-mesenchymal transition by reducing hnRNPM expression [69]. YY1 can also silence tumor suppressor genes, such as XAF1 in PCa [70]. In summary, YY1 is a recognized prostate cancer driver [66] and different complexes in which YY1 takes part can induce activation or repression of gene expression, including also AR-YY1-mediated PSA transcription [102], which we found is also regulated by HMGB1 and HMGB2 silencing. HOXA10 is upregulated in PCa [31], and inverse correlations between HOXA10 expression and Gleason pattern, Gleason score, and pathological stage are found [32], although downregulation of HOXA10 gene expression may enhance lipogenesis to promote PCa cell growth and tumor progression to the castration-resistant stage [103]. Silencing of HOXA10 expression in PC-3 cells by iRNA decreased proliferation rates, whereas HOXA10 overexpression had the opposite effect [31]. Physical interaction between these PCa-associated proteins and HMGB proteins has not previously been described, and our results therefore show that there is a connection between HMGB1 and HMGB2 functions and those of their binding partners in PCa.

Considering that HMGB1, HMGB2, and a subset of their interactome partners are upregulated in PCa, we silenced HMGB1 and HMGB2 and analyzed the mRNA levels of a group of randomly selected partners in PC-3 cells (Figure 6). The data show that HMGB1 and HMGB2 control the expression of them, which might contribute to the orchestrated action of all these proteins in PCa. HMGB2 activates many of the tested targets, but unexpectedly, HMGB1 has the opposite effect. One can propose several reasons to explain upregulation of targets in these circumstances. Data from the genotype-tissue expression (GTEx) project [104] indicates that, although both HMGB1 and HMGB2 are upregulated in PCa versus noncancerous cells, the relative increase is higher for HMGB2 (×1.5) than HMGB1 (×1.3); this could explain the increased expression of several of their targets, assuming that positive regulation caused by HMGB2 predominates over negative regulation caused by HMGB1 during the onset of PCa. Alternatively, differential interaction of HMGB1 or HMGB2 with their different nuclear partners, the transcript factors detected in our Y2H analysis being among them, might condition their positive or negative regulatory roles on the expression of specific genes.

Clinically, a high frequency of CNA of the genes encoding the identified proteins is associated with the most aggressive forms of PCa: small cell neuroendocrine carcinoma (SCNC) or castration-resistant PCa (Figure 3). Their gain or amplification in the genome of the cancerous cells are positively correlated to a lesser disease-free period for PCa patients (Figure 4). The mRNA levels of a subset of these proteins are also higher in metastases than primary tumors (Figure 5). In conclusion, the set of proteins detected though our HMGB1-HMGB2 Y2H analysis are associated with the most aggressive cases of PCa. Although the PSA-based test is routinely employed for screening of PCa, it has resulted in overdiagnosis and overtreatment of nonaggressive cancers, thus reducing the quality of life of patients.

**Figure 7:**
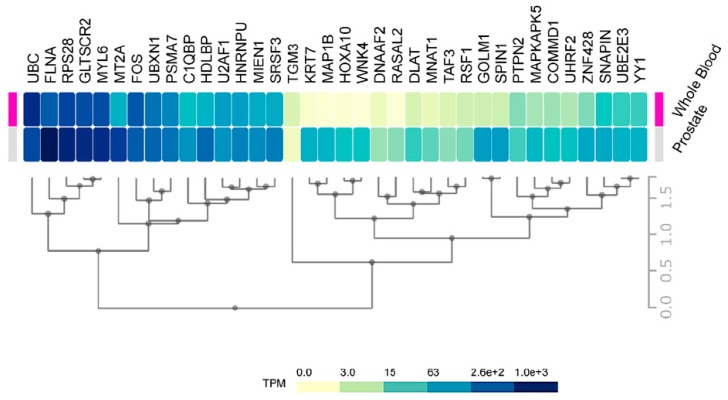
Prostate and blood levels of HMGB1 and HMGB2 interactome targets in healthy men: Expression of HMGB1 and HMGB2 interactome targets in prostate tissue and whole blood in healthy men. Data were directly obtained and processed from the GTEx Project through Expression Atlas, an integrated database of gene and protein expression in humans, animals, and plants [105], accessed through https://www.ebi.ac.uk/gxa/experiments/E-MTAB-5214/Results.

Consequently, an improvement is necessary in the initial stages to discriminate between high-risk from low risk cancers. Our data on HMGB1 and HMGB2 interactome targets, considering their correlation to high aggressiveness and bad prognosis, is a good starting point to develop new serum protein panels for improvement of PCa diagnosis. Indeed, FLNA has already been proposed in a clinical validated PCa biomarker panel in serum [74]. PSMA7 was also proposed as a PCa biomarker [55], and KRT7 is included in a whole blood mRNA 4-gene androgen regulated panel for PCa diagnosis [33]. Considering the relative expression levels of our HMGB1 and HMGB2 interactome targets in noncancerous cells or in blood of health subjects differ quite notably (Figure 7), one might anticipate that more sensitive analyses could be carried out using as biomarkers those proteins that are usually lowly expressed in noncancerous cells; thus, their levels are also low in the blood of healthy individuals. For instance, FLNA reported as a possible biomarker [74] is one of the 50 proteins most strongly expressed in normal prostate, and high levels are also detected in the blood of healthy individuals, whereas other detected HMGB1 or HMGB2 interactome targets in our study, e.g., DNAAF2, GOLM1, or TGM3, are in the lowest rank of detection in noncancerous samples and their increase should become more discriminatory.

## 4. Materials and Methods

### 4.1. Biological Materials

PC-3 is an androgen-independent cell line derived from a bone metastasis [106]. The human PCa PC-3 cell line, regularly validated by DNA typing, was obtained from the American Type Culture Collection ATCC and grown in Roswell Park Memorial Institute RPMI-1640 media, supplemented with 10% heat-inactivated fetal bovine serum and 1% penicillin-streptomycin (Thermo Fisher Scientific, Inc. Waltham, MA, USA). Cells were cultured at 37 °C in 5% CO_2_ in air in a humidified incubator. RNA from PCa tissue, isolated after radical prostate resection of a 66-year-old man diagnosed with adenocarcinoma (Gleason score 6) and not previously treated with radiotherapy or chemotherapy, was obtained from Biobanco de Andalucía (SPAIN).

### 4.2. Yeast Two Hybrid Methodology 

*Sacchacomyces cerevisiae* strains were Y187 (*MATα, ura3-52, his3-200, ade2-101, trp1-901, leu2-3, 112, gal4Δ, gal80Δ, met-*, and *URA3::GALuas-GAL1TATA-LacZ MEL1*) and Y2HGold (*MATa, trp1-901, leu2-3, 112, ura3-52, his3-200, gal4Δ, gal80Δ, LYS2::GAL1uas-GAl1TATA-His3, GAL2uas-Gal2TATA-Ade2 URA3:: MEL1UAS-Mel1TATA*, and *AUR1-C MEL1*).

Total RNA from the PC-3 cell line obtained from the supplier (Sigma-Aldrich, Saint Louis, MO, USA) and RNA from PCa tissue (Biobanco de Andalucía, Spain) were used to construct cDNA libraries. HMGB1 and HMGB2 interacting partners were identified using Matchmaker Gold Yeast 2-Hybrid System (Clontech, Fremont, CA, USA). Library construction, bait construction, and Yeast 2-Hybrid library screening were done according to the Takara Bio USA Matchmaker^®^ Gold Yeast 2-Hybrid System manual. In brief, the baits were cloned as fusions to the GAL4 activation domain in the plasmid pGBKT7-AD and used to transform the yeast haploid strain, Y187. cDNA libraries prepared from RNA extracted from PC-3 cells and PCa cancerous tissue were included as fusions to the GAL4 DNA-binding domain in the plasmid pGBKT7-BD and were used to transform the yeast haploid strain, Y2HGold. RNAs from human samples used to prepare the Y2H libraries were obtained from Biobanco de Andalucía (Spain). RNA was extracted from frozen tissue sections in OCT (Optimal Cutting Temperature) compound, using the Qiacube robot from QIAGEN, based on ion-exchange columns with a silica membrane. RNA was obtained with the miRNeasy mini-kit from QIAGEN that allows recovery of both total RNA and miRNAs. The samples were finally treated with RNase-free DNAase from QIAGEN. The RNA was quantified at 260 nm and 280 nm by spectrophotometry using Infinite F200 equipment of TECAN with a Nanoquant plate. Finally, the integrity of the samples was evaluated by AGILENT 2200 Tape Station apparatus, with the RIN (RNA Integrity Number) parameter being >8. Efficiency in the constructions of libraries was in the range recommended in the kit (all libraries guaranteed to have >1 × 10^6^ independent clones). As a previous control, we confirmed that our baits (HMGB1 and HMGB2) do not autonomously activate the reporter genes in Y2HGold in the absence of a prey protein. Bait and prey fusion proteins are each expressed in different haploid yeast strains that can form diploids. The diploid yeast cell expresses both proteins, and when fusion proteins interact, the transcriptional activator GAL4 is reconstructed and brought into proximity to activate transcription of the reporter genes. For diploid formation, 1 mL of concentrated bait culture was combined with 1 mL of library culture and incubated overnight with slow shaking. A drop of the culture was checked under a phase-contrast microscope (40×) to confirm the existence of zygotes before plating on diploid-selective media. Diploids were tested for expression of the reporter genes in selective media. To reduce the appearance of false positives, a screening based on three different independent markers (ADE2, HIS3, and MEL1) was selected. pGBKT7-BD plasmids carrying the preys were rescued from confirmed positive diploids, and DNA was used to transform *E. coli*. The inserts were sequenced with primer T7 (5′-TAATACGACTCACTATAGGG-3′). Sequences were used for homology searches with BlastN and BlastX at the National Center for Biotechnology Information NCBI (https://blast.ncbi.nlm.nih.gov/) and proteins in the database matching the queries annotated as positives.

### 4.3. Expression Analysis by Quantitative Polymerase Chain Reaction (RT-qPCR)

Individual analyses of gene expression were carried out as follows. RNA samples were retro-transcribed into cDNA and labeled with the *KAPPA SYBR FAST* universal one-step qRT-PCR kit (Kappa Biosystems, Inc, Woburn, Massachusetts, USA). The primers for qPCR are shown in Table S3. Reaction conditions for thermal cycling were 42 °C for 5 min, 95 °C for 5 s, 40 cycles of 95 °C for 3 s, and finally 60 °C for 20 s. ECO Real-Time PCR System was used for the experiments (Illumina, Inc., San Diego, California, USA), and calculations were made by the 2^−ΔΔCt^ method [107]. Student’s test was used to check the statistical significance of differences between samples (*p* < 0.05). The relative mRNA levels of the experimentally selected genes (target genes) were calculated by referring to the mRNA levels of the housekeeping gene, encoding glyceraldehyde phosphate dehydrogenase (GAPDH), which had been verified as being expressed constitutively under the assay conditions. For valid quantification using the 2^−ΔΔCt^ method, it is crucial that target and housekeeping PCR amplification efficiencies are approximately equal: we therefore verified that the efficiencies of the 2 PCR reactions differed by <10%. At least 2 independent biological replicas and 3 technical replicas of each of them were made for all the experiments.

### 4.4. Immunoprecipitation

One hundred µl of Protein G Plus-Agarose immunoprecipitation-reagent (Santa Cruz Biotechnology, Dallas, TX, USA) were coupled with 4 µg of anti-HMGB1 antibody (sc-74085; Santa Cruz Biotechnology) or anti-mouse antibody (Molecular Probes, A10534) in phosphate buffered saline (PBS) for 1 h at 4 °C with rotation. PC-3 cells were lysed in 20 mM Tris/HCl, 150 mM, 1% Triton X-100, 1× phenylmethylsulfonyl fluoride (PMSF), and protease inhibitor cocktail (Sigma-Aldrich, Saint Louis, MO, USA) and incubated for 30 min at 4 °C with rotation. Total protein (500 µg) was incubated with the antibody agarose beads overnight and eluted by incubation in 1× lithium dodecyl sulfate LDS loading buffer containing 350 mM β-mercaptoethanol at 95 °C for 10 min. Mass spectrometry and data analysis were done as previously described [26].

### 4.5. Western Blot Analysis

Protein samples were run on 10% SDS-PAGE gels at 80 V for 20 min followed by 200 V for 45–60 min. Proteins were transferred onto a polyvinylidene fluoride (PVDF) membrane at 0.2 A for 1 h. Membranes were blocked by incubating with 5% non-fat dry milk for 1 h at room temperature (RT) and then incubated with primary antibodies, anti-HMGB1 (sc-74085; Santa Cruz Biotechnology) or anti-Cytokeratin 7 (ab181598; Abcam, Cambridge, UK) in phosphat*e*-buffered *s*aline with 0.1% Tween 20^®^ detergent PBST overnight at 4 °C. After incubation with the corresponding horseradish peroxidase-conjugated secondary antibody, enhanced chemiluminescence for high sensitivity and long-lasting signal (ECL) Anti-mouse IgG (NXA931 from GE Healthcare Sciences, Chicago, IL, USA) or ECL Anti-rabbit IgG (NA934 from GE Healthcare Sciences, Chicago, IL, USA), protein bands were detected using LuminataTMCrescendo Western HRP Substrate (Millipore Corporation, Burlington, MA, USA) and a ChemiDocTM imager (Bio-Rad laboratories Hercules, CA, USA).

### 4.6. Immunofluorescence and Confocal Microscopy

Cells were plated in 6-well plates, each containing 4 sterile 13-mm glass coverslips. When 80% confluent, cells were fixed in 4% paraformaldehyde in PBS for 15 min at RT. Cells were washed 3 times with PBS (137 mM NaCl, 2.7 mM KCl, 10 mM Na_2_HPO_4_, and 2 mM KH_2_PO_4_) and finally treated with 0.1% Triton/PBS for 15 min at RT. They were then blocked in 1% bovine serum albumin (BSA) in PBS for 1 h at RT. Primary antibodies, anti-HMGB1 (sc-74085; Santa Cruz Biotechnology) or anti-Cytokeratin 7 (ab181598; Abcam, Cambridge, UK) were diluted in 1% BSA in PBS. Cells were incubated with the corresponding primary antibodies overnight at 4 °C, followed by 3 washes with PBS and staining with the secondary antibodies, modified with Alexa Fluor 488 and 568 (Invitrogen, Carlsbad, CA, USA) previously diluted in 1% BSA in PBS for 1 h at RT in the dark. For nuclear staining, after secondary antibody incubation, wells were washed 3 times and stained with Hoechst (Life Technologies, Carlsbad, CA, USA) for 5 min at RT in the dark. Cells were washed once with PBS and once with sterile distilled water. Each coverslip was mounted on a clean slide using ProLong™ Gold Antifade Mountant (Invitrogen). After drying, the slides were stored at 4 °C in the dark until they were examined by confocal microscopy (Nikon A1R). Meander’s correlation coefficient was calculated using Nis Elements software from Nikon.

### 4.7. HMGB1 and HMGB2 Silencing by siRNA

The PC-3 cell line was transfected with small interfering (si)RNA oligonucleotides using Lipofectamine 2000 (Invitrogen). siRNA and Lipofectamine 2000 were each diluted separately with Opti-MEM (Gibco), mixed together, and incubated for 5 min at RT. The mixture was added to cells plated in 3 mL RPMI 1610 medium (final concentration of siRNA, 50 nM). Cells were collected at 48 h post transfection for further analysis. The following siRNAs (Life Technologies) were used for the silencing of each gene: s20254 Silencer Select for HMGB1, s6650 for HMGB2, and AS02A5Z3 for the siRNA negative control.

Total RNA was extracted from different conditions (siHMGB1, siHMGB2, and siCtrl#2) of the PC-3 cell line using GeneJET RNA Purification Kit (#K0731, Thermo Scientific). The remaining DNA was removed by incubating with DNase I, RNase-free (#EN0521, Thermo Scientific). DNA-free RNA was finally purified using GeneJET RNA Cleanup and Concentration Micro Kit (#K0842, Thermo Scientific). qPCR reactions were run in triplicate in an Eco Real-Time PCR System (Illumina) using 1 ng per reaction. PC-3 lysates of each condition were extracted with lysis buffer (50 mM Tris-HCl pH 8, 150 mM NaCl, 0.1% NP40, 1 mM ethylenediaminetetraacetic acid disodium salt (EDTA), and 2 mM MgCl_2_), and protein concentration was quantified using Bradford Reagent (Bio-Rad). Protein samples of 25–40 µg were loaded for western blotting. PVDF membranes were incubated overnight at 4 °C with primary antibodies, anti-HMGB1 (ab18256, Abcam), anti-HMGB2 (ab67282, Abcam), or anti-α-tubulin (sc53646, Santa Cruz Biotechnology).

### 4.8. Heat Maps

Heat maps from change-fold ratios (Figure 5 and Figure 6) were drawn with Heatmapper (http://heatmapper.ca/expression/), using complete linkage as clustering method and Euclidean distance as the measurement method [108].

### 4.9. Statistical Analysis 

Analyses were carried out using GraphPad Prism 6 (GraphPad Software, San Diego, CA, USA). Continuous variables were expressed as mean ± SE. Relative gene expression assays were tested using independent t-tests. A 2-tailed *p*-value test was used with *p* < 0.05 considered significant.

## 5. Conclusions

We have carried out the first HMGB1/HMGB2 interactome approach in prostate cancer (PCa) using both the PC-3 cell line and adenocarcinoma tissue. Gene or protein expressions of the majority of targets are dysregulated in PCa, and functional relationships between these proteins and PCa had also previously been confirmed by different laboratories using different models and technical approaches. We have shown by interference analysis that several HMGB1 and HMGB2 partners are regulated by HMGB1 and HMGB2 themselves, which might contribute to the coordination of their cellular action in PCa. Copy number alterations in the detected HMGB1 and HMGB2 partners are associated with aggressive forms of PCa and a poor prognosis. These characteristics can potentially be used as discriminatory biomarkers between high and low risk patients.

## Figures and Tables

**Table 1:** Proteins identified in the high mobility group box B 1 (HMGB1) yeast 2-hybrid (Y2H) approach interactome in PC-3 cells.

Gene (Aliases)	Uniprot Code	N	A	Biological Function	Previous References to Prostate Cancer (PCa)
DLAT (DLTA)	P10515	1	221–420	Dihydrolipoamide-acetyltransferase (DLAT) in pyruvate dehydrogenase complex control of mitochondrial energetic metabolism [27].	Enzymatic activity at the basal level is significantly higher in prostate cancer cells compared to benign prostate cells [28].
DNAAF2 (KTU)	Q9NVR5	1	436–621	Cytoskeletal component [29].	Not previously reported
HDLBP (HBP, VGL)	Q00341	1	152–374	HDLBP drives cell proliferation [30].	Not previously reported
HOXA10 (HOX1H)	P31260	1	*	Transcriptional control	HoxA10 is highly expressed in PCa cells and tissues and is involved in cancer cell proliferation [31,32].
KRT7 (SCL)	P08729	11	109–301	Cytoskeletal component	KRT7 circulating mRNA was identified in blood samples from a cohort of metastatic PCa patients [33].
MNAT1	P51948	1	*	MAT1, encoded by MNAT1, binds to p53 and mediates p53 ubiquitin-degradation through MDM2, increases cell growth, and decreases cell apoptosis [34].	Not previously reported
SPIN1	Q9Y657	1	130–337	Chromatin reader; promotes the expression of rRNA [35]	Not previously reported
UBE2E3	Q969T4	5	25–111	Control of transcription factor activity [36].	Not previously reported
UBXN1 (SAKS1)	Q04323	1	57–238	NF-κB can be negatively regulated by UBXN1 [37].	Not previously reported
UHRF2 (NIRF, RNF107)	Q96PU4	4	157–284	UHRF2 encodes a nuclear protein involved in cell-cycle regulation, and it is an important mediator of E2F1-induced cell death [38].	Not previously reported
ZNF428 (C19orf37)	Q96B54	1	109–188	Unknown	Not previously reported

N: redundancy in clone isolation; A: Sequenced region in clones, Aa relative to ATG; * noncoding sequence.

**Table 2:** Proteins identified in the the high mobility group box B 2 (HMGB2) Y2H interactome in PC-3 cells.

Gene (Aliases)	Uniprot Code	N	A	Biological Function	Previous References to Prostate Cancer (PCa)
C1QBP (GC1QBP, HABP1, SF2P32)	Q07021	3	1–187	Control of mitochondrial energetic metabolism. Promotes cell proliferation, migration, and resistance to cell death. [39].	Highly expressed in prostate cancer and is associated with shorter prostate-specific antigen relapse time after radical prostatectomy [40].
SNAPIN (BLOC1S7, SNAP25BP)	O95295	1	54–136	A SNARE-associated protein which binds Snap25 facilitating the vesicular membrane fusion process [41].	Involved in developing prostate adenocarcinoma in mice [41].
U2AF1 (U2AF35,)	Q01081	1	31–104	RNA splicing [42].	Highly expressed in PCa [43].
UHRF2 (NIRF, RNF107)	Q96PU4	1	20–169	UHRF2 encodes a nuclear protein involved in cell-cycle regulation and is an important mediator of E2F1-induced cell death [38].	Not previously reported
ZNF428 (C19orf37)	Q96B54	3	100–188	Unknown	Not previously reported

N: redundancy in clone isolation; A: Sequenced region in clones, Aa relative to ATG.

**Table 3:** Proteins identified in the HMGB1 Y2H interactome in primary tumor adenocarcinoma.

Gene (Aliases)	Uniprot Code	N	A	Biological Function	Previous References to Prostate Cancer (PCa)
c-FOS	P01100	2	27–184	Transcriptional regulation and control of cell growth and apoptosis. [44].	Expression is elevated in the prostate upon castration-mediated androgen withdrawal [44].
GOLM1	Q8NBJ4	1	236–376	PI3K-AKT-mTOR signaling [45].	Upregulated in PCa has oncogenic functions [45].
HNRNPU	Q00839	1	91–296	DNA and RNA binding [46].	Not previously reported
MAP1B	P46821	2	2187–2409	Vesicle formation; it can interact with p53 [47].	Not previously reported
MAPKAPK5	Q8IW41	1	1–95	Involved in mTOR signaling [48]; MAPKAPK5 has diverse roles in cell growth, programmed cell death, senescence, and motility [49].	Not previously reported
MIEN1	Q9BRT3	3	24–204	Regulator of cell migration and invasion [50].	MIEN1 increases invasive potential of PCa cells by NF-κβ-mediated downstream target genes [50].
MT2A	P02795	1	8–61	Binding to heavy metals [51].	MT2A is upregulated under hypoxia in PCa cell lines, PCa tissue, and residual cancer cells after androgen ablation therapy [52].
PSMA7 (PTPT)	O14818	1	173–248	PSMA7, a proteasome subunit, enhances AR transactivation in a dose-dependent manner [53] and inhibits the transactivation function of HIF-1A [54].	Proposed biomarker in PCa [55]
PTPN2	P17706	3	1–221	Tyrosine-specific phosphatase (TCPTP) negatively regulates STAT3 that is involved in cell growth and proliferation, differentiation, migration, and cell death or apoptosis [56].	Not previously reported
RASAL2	Q9UJF2	1	97–334	Tumor suppressor via RAS [57]	Not previously reported
RSF1	Q96T23	1	572–795	Chromatin remodeling factor necessary for p53-dependent gene expression in response to DNA damage [58].	RSF1 is overexpressed in PCa and contributes to prostate cancer cell growth and invasion [59].
SRSF3	P84103	2	1–164	Oncogenic splicing factor [60].	SRSF3 expression is induced by hypoxia in prostate cancerous cells [61].
TAF3	Q5VWG9	5	2–222	Transcriptional regulation; interacts with and inhibits p53 [62].	Not previously reported
TGM3	Q08188	1	480–693	Catalyze the irreversible cross-linking of peptide-bound glutamine residues to lysines or primary amines; involved in apoptosis [63].	Not previously reported
UBC	P0CG48	1	28–181	Unanchored-polyubiquitin has several roles in activation of protein kinases, and signaling	Not previously reported
WNK4	Q96J92	4	9–208	Regulates STE20-related protein kinases that function upstream of the MAPK pathways. [64].	Not previously reported
YY1	P25490	1	27–223	Transcriptional regulation [65].	Involved in PCa [65,66,67,68,69,70]
ZNF428	Q96B54	2	89–188	Unknown	Not previously reported

N: redundancy in clone isolation; A: Sequenced region in clones, Aa relative to ATG.

**Table 4:** Proteins identified in the HMGB2 Y2H interactome in primary tumor adenocarcinoma.

Gene (Aliases)	Uniprot Code	N	A	Biological Function	Previous References to Prostate Cancer
C1QBP (GC1QBP, HABP1, SF2P32)	Q07021	10	57–282	Control of mitochondrial energetic metabolism; promotes cell proliferation, migration, and resistance to cell death. [39].	Highly expressed in PCa and associated with shorter prostate-specific antigen relapse time after radical prostatectomy [40].
COMMD1	Q8N668	1	1–180	Regulates oxidative stress, NF-κB-mediated transcription, DNA damage response, and oncogenesis [71].	Degradation of COMMD1 and I-kappaB induced by clusterin enhances NF-κβ activity in prostate cancer cells. [72].
FLNA	P21333	5	106–366	A C-terminal fragment of FLNA co-localizes with the androgen receptor AR to the nucleus and downregulates AR function. [73].	FLNA has been clinically validated for better diagnosis of PCa [74]; regulated by miRNA205 [75].
MIEN1	Q9BRT3	4	1–116	Regulates cell migration and apoptosis [50].	Overexpressed in PCa cells. MIEN1 overexpression functionally enhances migration and invasion of tumor cells via modulating the activity of AKT [50].
MYL6	P60660	2	1–150	Regulatory light chain of myosin II; myosin II, expressed in non-muscle tissues, plays a central role in cell adhesion, migration, and division [76].	Not previously reported
NOP53 (GLTSCR2)	Q9NZM5	35	163–428	Cell cycle control; NOP53 translocates to the nucleoplasm under ribosomal stress, where it interacts with and stabilizes p53 and inhibits cell cycle progression [77].	Not previously reported
RPS28	P62857	1	8–52	Ribosome component; its decrease blocks pre-18S ribosomal RNA processing, resulting in a reduction in the assembly of 40S ribosomal subunits [78].	Not previously reported

N: redundancy in clone isolation; A: Sequenced region in clones, Aa relative to ATG

## Supplementary Materials

The following are available online at https://www.mdpi.com/2072-6694/11/11/1729/s1, Table S1: Association of proteins that interact with HMGB1 or HMGB2 to cancer hallmarks, Table S2: Prostate cancer studies available through cBioportal, Table S3: List of oligonucleotides used in this work. Figure S1: Y2H triple screening by 3 independent selection markers. Image S1: Blots corresponding to Figure 2a. Image S2: Blots corresponding to Figure 6a.
