# Supplementary material for: Characterization of HMGB1/2 Interactome in Prostate Cancer by Yeast Two Hybrid Approach: Potential Pathobiological Implications
Source: Cancers (Basel). 2019 Nov 5;11(11):1729. doi: 10.3390/cancers11111729 (PMC6895793; doi:10.3390/cancers11111729)
Supplement: Supplementary file 1 [file cancers-11-01729-s001.pdf]

cancers

MDPI

Article

Characterization of HMGB1/2 Interactome in Prostate Cancer by Yeast Two Hybrid Approach: Potential Pathobiological Implications

Aida Barreiro-Alonso [1,†], María Cámara-Quílez [1,†], Martín Salamini-Montemurri [1], Mónica Lamas-Maceiras [1], Ángel Vizoso-Vázquez [1], Esther Rodríguez-Belmonte [1], María Quindós-Varela [2], Olaia Martínez-Iglesias [3], Angélica Figueroa [3] and María-Esperanza Cerdán [1,*]

Table S1. Association of proteins that interact with Hmgb1 or Hmgb2 to cancer hallmarks.

Cancer Hallmark	Protein	Model	Reference
GENOMIC INSTABILITY AND MUTATIONS/ CHANGES IN TELOMERASE ACTIVITY	MAP1B	Colorectal cancers	[109]
	NOP53	Liver adenocarcinoma (SKHep1) and glioblastoma (T98G) cell lines	[110]
	RSF1	Ovarian cells	[111]
	SRSF3	Ovarian cancer	[112]
SUSTAINING PROLIFERATIVE SIGNALLING/ CELL PROLIFERATION	C1QBP	Breast cancer	[54,113]
	cFOS	Bladder cancer	[114]
	cFOS	Breast cancer	[115]
	DLAT	Gastric Cancer	[116]
	FLNA	Human melanoma (A7), Prostate cancer (PC3) cell lines	[117]
	GOLM1	Hepatocellular carcinoma	[118]
	GOLM1	Breast cancer	[119]
	GOLM1	Lung proliferation	[120]
	GOLM1	Prostate cancer	[82]
	Hox-A10	Prostate cancer cell line PC-3	[58]
	Hox-A10	Ovarian cancer cell lines	[121,122]
	NOP53	Breast cancer	[123]
	PSMA7	Cervical cancer	[124]
	PTPN2	Epithelial carcinogenesis	[125]
	RASAL2	hepatocellular carcinoma	[126]
	RSF1	Prostate cancer	[95]
	RSF1	Nasopharyngeal carcinoma	[127]
	SPIN1	Glioma	[128]
	TGM3	Esophageal cancer	[129]
	UBE2E3	Retinal pigment epithelial (RPE)	[130]
	Vigilin	Hepatocellular carcinomas	[131]
	WNK4	Kidney	[100]
EVADING GROWTH SUPPRESSORS/ CELL CYCLE CONTROL	C1QBP	Prostate cancer	[48]
	COMMD1	Neuroblastoma	[132]
	FLNA	Pulmonary neuroendocrine tumors	[133]
	NOP53	Lung cancer A549 and H1299 cell lines	[107]
	RSF1	Nasopharyngeal carcinoma	[127]
	SPIN1	Glioma	[128]
	Vigilin	Liver carcinoma HepG2 cells	[134]
RESISTING CELL DEATH APOTOSIS /PHAGOCITOSIS IMBALANCE (continued)	FLNA	bladder carcinoma	[117]
	GOLM1	Prostate cancer	[82]
	Hox-A10	Epithelial ovarian cancer cell lines	[122]
	MAP1B	Melanoma and colon carcinoma cell lines	[135]
	MAPKAPK5	Hepatocellular carcinoma	[136]

Hallmark	Protein	Model	Reference
	MNAT1	Colorectal cancer	[73]
	MT2A	Colorectal cancer	[137]
	PSMA7	Cervical cancer	[124]
	PSMA7	Colorectal cancer	[138]
	RSF1	Nasopharyngeal carcinoma	[127]
	SPIN1	Glioma	[128]
	SRSF3	oral squamous cell carcinoma	[139]
	**Protein**	**Model**	**Reference**
	SRSF3	Human colon adenocarcinoma and Human osteosarcoma cell lines	[140]
	SRSF3	Ovarian cancer	[141]
	TGM3	Esophageal cancer	[129]
	U2AF1	Ba/F3 cells<br>Lung adenocarcinoma cell lines	[80,142]
	YY1	Several models	[143]
ACTIVATING INVASION AND METASTASIS	C1QBP	Breast cancer metastasis in mouse model	[144]
	COMMD1	Mouse melanoma cells	[145]
	FLNA	Human melanoma (A7), prostate cancer (PC3) cell lines	[117]
	GOLM1	Hepatocellular carcinoma	[118]
	GOLM1	Breast cancer	[119]
	GOLM1	Cervical cancer	[146]
	GOLM1	Prostate cancer	[82]
	Hox-A10	Ovarian cancer cell line ES-2	[121]
	MAPKAPK5	Hela Cells	[86]
	MIEN1	Breast cancer	[147]
	MIEN1	Prostate cancer	[148]
	NOP53	Endometrial cancer	[149]
	RASAL2	Colorectal cancer	[150]
	RASAL2	hepatocellular carcinoma	[126]
	RASAL2	Lung cancer	[151]
	RSF1	Prostate cancer	[95]
	RSF1	Non-small cell lung cancer	[152]
	SRSF3	HeLa cells	[153]
	TGM3	Esophageal cancer	[129]
	UHRF2	Gastric cancer cell lines	[154]
	Vigilin	Hepatocellular carcinomas	[131]
	YY1	Cancer cell lines	[155]
DEREGULATED CELLULAR ENERGETICS / CARBOHYDRATE METABOLISM REPROGRAMMING	C1QBP	Human embryonic kidney HEK293T cells	[69]
	DLAT	Gastric cancer cell lines	[116]
	SRSF3	Colon cancer cells	[156]

Table S2. Prostate cancer studies available through cBioportal.

cBioportal Name	References	Samples	Sequenced	Cna	RNA Seq	DNA Arrays	Survival Studies
Metastatic prostate adenocarcinoma (mctp, nature 2012)	[40]	61	61	61			
Metastatic prostate adenocarcinoma (su2c/pcf dream team, pnas 2019)	[41]	444	444	444			Yes
Metastatic prostate cancer (su2c/pcf dream team, cell 2015)	[42]	150	150	150	118		
Neuroendocrine prostate cancer (multi-institute, nat med 2016)	[43]	114	114	114	49		
Prostate adenocarcinoma (broad/cornell, cell 2013)	[29]	57	57	56			
Prostate adenocarcinoma (broad/cornell, nat genet 2012)	[30]	112	112	109			
Prostate adenocarcinoma (cpc-gene, nature 2017)	[31]	477	477	0			
Prostate adenocarcinoma (eururol, 2017)	[32]	65	65	65			
Prostate adenocarcinoma (fred hutchinson crc, nat med 2016)	[33]	176	141	149			
Prostate adenocarcinoma (mskcc, cancer cell 2010)	[34]	216	103	194		150	Yes
Prostate adenocarcinoma (mskcc/dfci, nature genetics 2018)	[35]	1013	1013	1013			
Prostate adenocarcinoma (tcga, cell 2015)	[157]	333	333	333	290		
Prostate adenocarcinoma (tcga, pancancer atlas)	[36]	494	494	489	493		
Prostate adenocarcinoma (tcga, provisional)		499	499	492	498		Yes
Prostate adenocarcinoma cna study (mskcc, pnas 2014)	[37]	104	0	104			Yes
Prostate adenocarcinoma organoids (mskcc, cell 2014)	[38]	12	12	0	10		
Prostate cancer (mskcc, jco precis oncol 2017)	[39]	504	504	504			
The metastatic prostate cancer project (provisional, december 2018)		19	19	19			

Table S3. qPCR primer list.

Gene Name	Sequence	TM (°C) *	Hybridization Site **	Amplicon Size (bp)
***AGER* (RAGE)**	F: 5′-TGTGTGGCCACCCATTCC-3′	60.47	901–918	109
	R: 5′-CTGATCCTCCCACAGAGCC-3′	59.75	991–1009	
DLAT	F: 5′-AACAGCGTGACTACAGGGTATG-3′	60.68	795–816	101
	R: 5′-CCCAAAAGCTGCAGCAGTAAG-3′	60.64	875–895	
FLNA	F: 5′-ACAGTGTCAATCGGAGGTCAC-3′	60.58	4942–4961	118
	R: 5′-TGCACGTCACTTTGCCTTTG-3′	60.47	5040–5059	
CFOS	F: 5′-GGGATAGCCTCTCTTACTACCAC-3′	59.77	71–93	124
	R: 5′-GTGACCGTGGGAATGAAGTTG-3′	60.04	174–194	
GAPDH	F: 5′-CCTCCTGCACCACCAACTG-3′	61.18	449–467	102
	R: 5′-TGGCAGTGATGGCATGGA-3′	59.50	533–550	
HMGB1	F: 5′-TCAAAGGAGAACATCCTGGCC-3′	60.59	338–358	87
	R: 5′- GCTTGTCATCTGCAGCAGTGTT-3′	62.54	403–424	
HMGB2	F: 5′-GAGCAGTCAGCCAAAGATAAACAA-3′	60.37	403–426	111
	R: 5′-TCCTGCTTCACTTTTGCCCTT-3′	61.01	493–513	
HOXA10	F: 5′-CTCCCACACTCGCCATCTC-3′	60.43	1341–1359	187
	R: 5′-CAAACCCAGCCCAGTCAGG-3′	61.19	1509–1527	
***KLK3* (PSA)**	F: 5′-ACCCTGGCAGGTGCTTG-3′	60.08	108–124	116
	R: 5′-GCAAGATCACGCTTTTGTTCCT-3′	60.61	202–223	
KRT7	F: 5′-TGAATGATGAGATCAACTTCCTCAG-3′	59.25	653–677	75
	R: 5′-TGTCGGAGATCTGGGACTGC-3′	61.91	708–727	
MAP1B	F: 5′-ACATCTTGGAACCTCCCACATC-3′	60.62	731–752	98
	R: 5′-TGCAAACAAGGCAGAATCGC-3′	60.61	809–828	
MIEN1	F: 5′-TTGGGGGCAGGAGAGAGAC-3′	61.21	519–537	107
	R: 5′-TTACCGAGGCGAAGAGTGG-3′	59.68	607–625	
MNAT1	F: 5′-TGTGCGGACACACTCTCTGTGAAA-3′	65.09	74–97	145
	R: 5′-TCAACCTCCTTGTCAACAGTGGGA-3′	64.37	195–218	
MT2A	F: 5′-AAAGGGGCGTCGGACAAG-3′	60.54	223–240	118
	R: 5′- GGTCACGGTCAGGGTTGTAC-3′	60.90	321–340	
NOP53	F: 5′-ACCAGTTCCTGGAAGACGTG-3′	60.19	182–201	109
	R: 5′-CCTTTTTCCTTGGAGCCAG-3′	56.66	272–290	
PMEPA1	F: 5′-AAGAGGAGTGAGAGGAAGGC-3′	59.02	929–948	110
	R: 5′-GCTTGTGCATTCAGACCAGA-3′	59.05	1019–1038	
SNAPIN	F: 5′-TGACAACCTAGCCACAGAACTG-3′	60.81	174–195	97
	R: 5′-TCGCCGGGCATTAAGTAGC-3′	60.80	252–270	
UBE2E3	F: 5′-AAGGTTACTTTCCGCACCAG-3′	58.69	376–395	102
	R: 5′-AATAGTCAAAGCGGGACTCCA-3′	59.69	457–477	
UHRF2	F: 5′-GGACCTTCCAATCAGCCATC-3′	58.90	316–335	99
	R: 5′-GACATCTCTGGCATCCACCA-3′	60.04	395–414	
YY1	F: 5′-AGCGGCAAGAAGAGTTACCTC-3′	60.65	538–558	103
	R: 5′-TCTTGATCTGCACCTGCTTCTG-3′	61.20	619–640	
ZNF428	F: 5′-CCCGAGCATTCCTCTGATTC-3′	58.70	79–98	95
	R: 5′-TCGTCAGTGGTCTCCTCTTC-3′	59.04	154–173	

* Tms were calculated using MFEprimer3.0 (http://mfeprimer.igenetech.com/) ** Hybridization sites are referenced to the starting codon (cDNA) for each gene (Ensembl database).

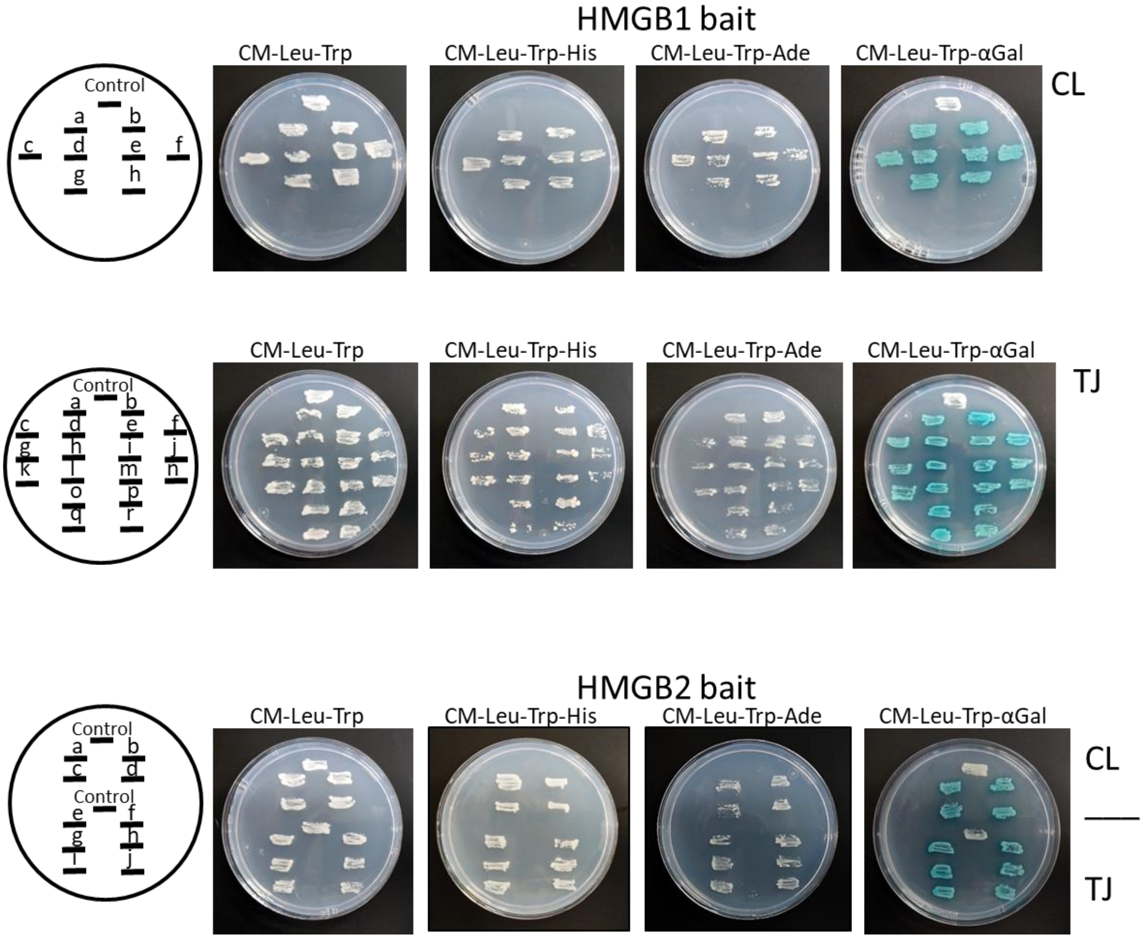

Figure S1. Re-screening of Y2H candidates and triple selection for positive growth in media without histidine, adenine or reporting alfa-galactosidase activity. Single colonies which were positive in the first screening as described in Materials and Methods were re-plated on selective media. CL, candidates from PC-3 libraries; TJ, candidates from PCa tissue libraries. Control represent cells transformed with the corresponding bait plasmid and an empty prey-plasmid. Upper panel: a, DLAT; b, DANNAF; c, HDLBP; d, HOXA10; e, KRT7; f, UBE2E3; g, UHRF2; h, ZNF428. Middle panel: a, FOS; b, GOLM1; c, HNRHPU; d, MAP1B; e, MAPKAPK5; f, MIEN1; g, MT2A; h, PSMA7; i, PTPN2; j, RASAL2; k, RSF1; l, SRSF3; m, TAF3; n, TGM3; o, UBC; p, WINK4; q, YY1; r, ZNF48. Lower panel: a, C1QBP; b, SNAPIN; c, UHRF2; d, ZNF428; e, C1QBP; f, COMMD1; g, FLNA; h, MIEN1; i, NOP53; j, RSP28.

Image S1. Whole Blots accompanying Figure 2A

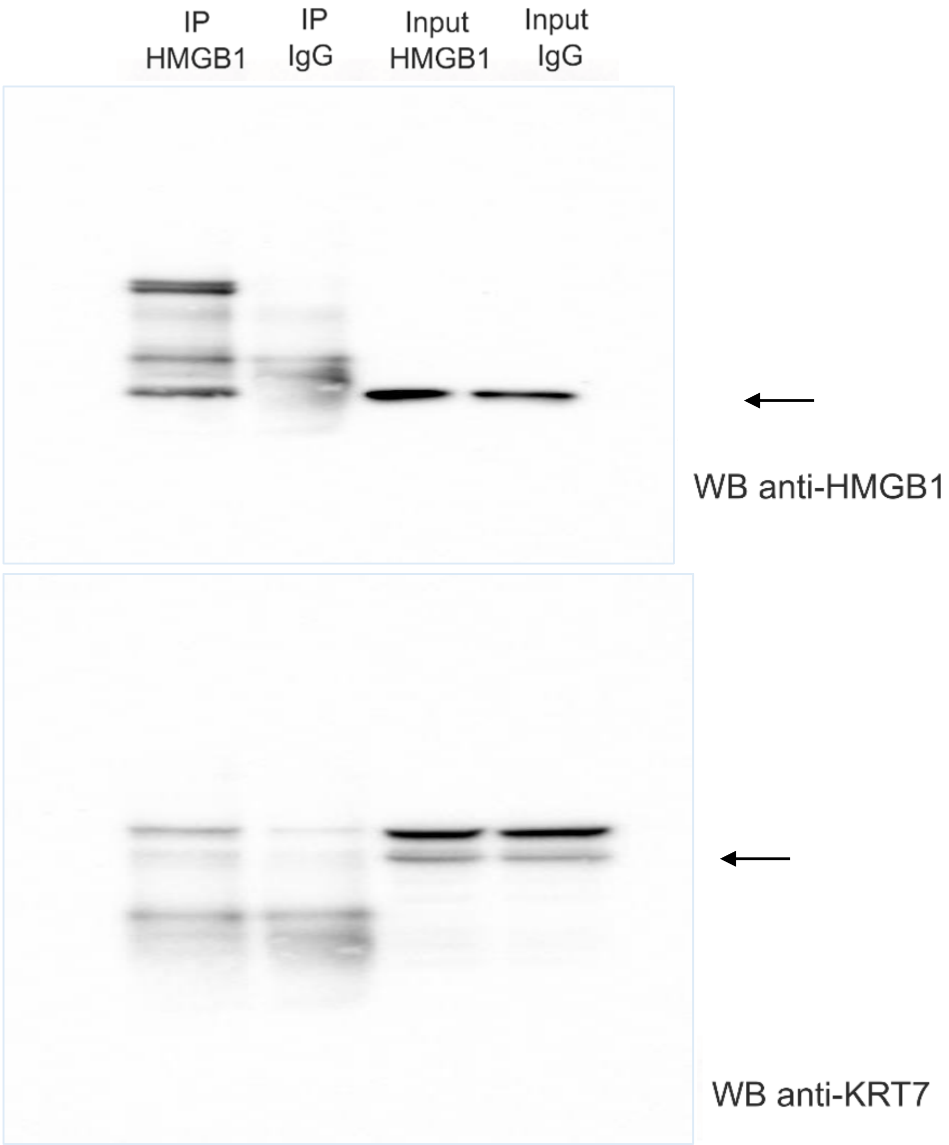

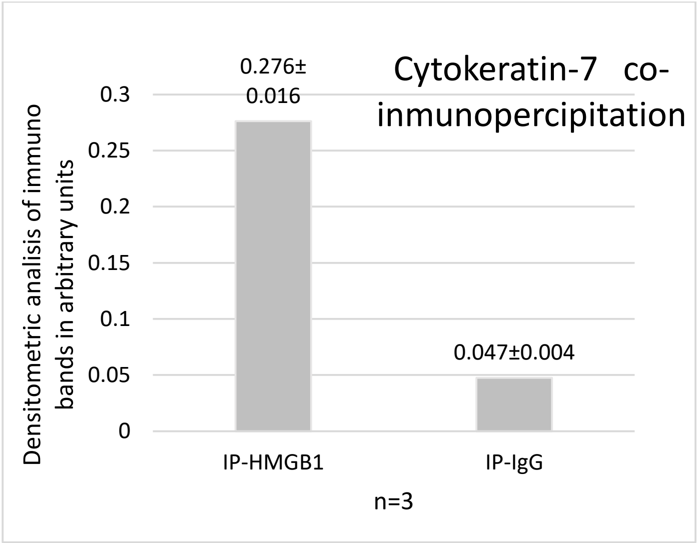

Image S2. Whole Blots accompanying Figure 6.

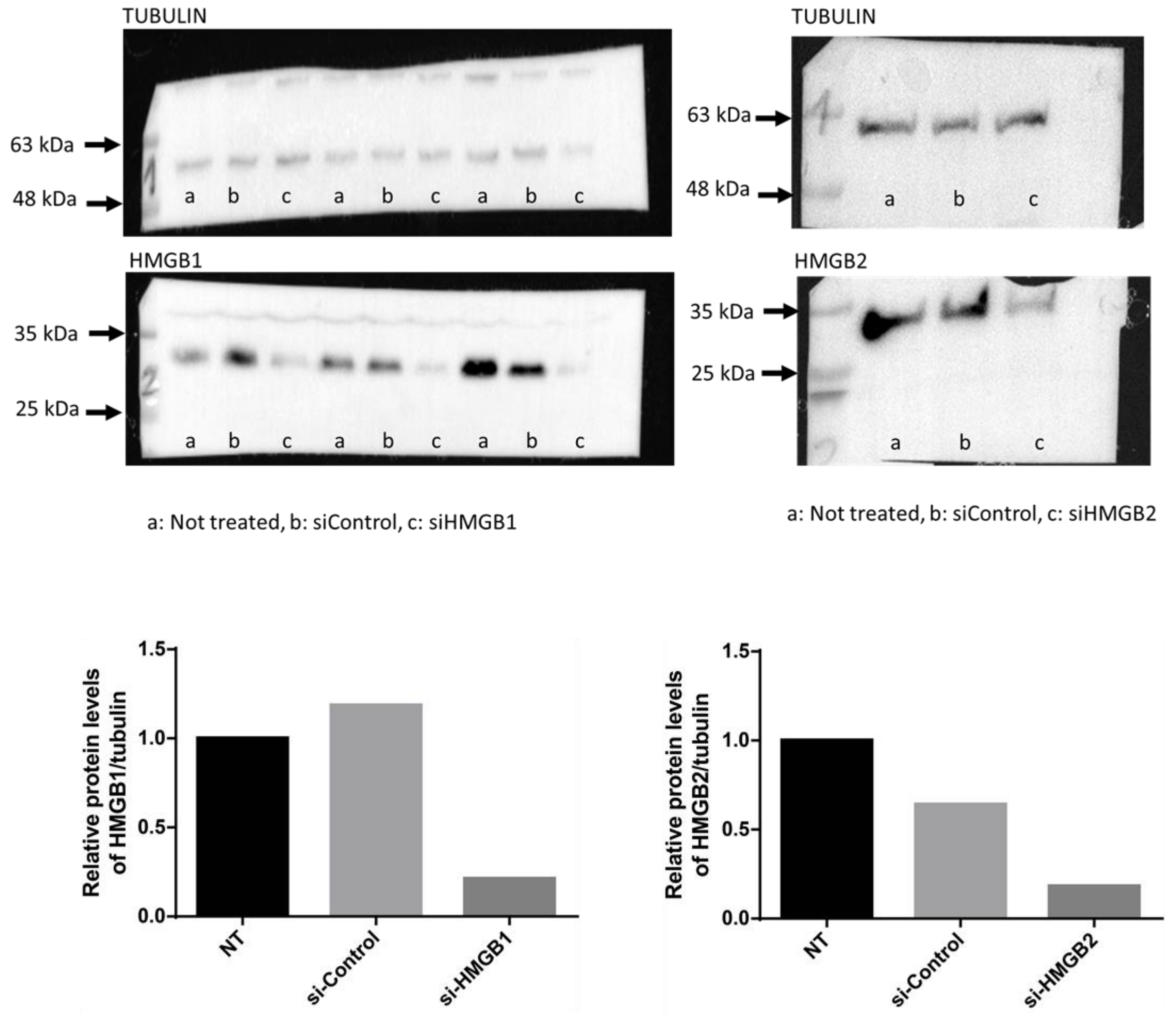

References

1. Catena, R.; Escoffier, E.; Caron, C.; Khochbin, S.; Martianov, I.; Davidson, I. HMGB4, a Novel Member of the HMGB Family, is Preferentially Expressed in the Mouse Testis and Localizes to the Basal Pole of Elongating Spermatids. *Biol. Reprod.* **2009**, *80*, 358–366.
2. Pusterla, T.; de Marchis, F.; Palumbo, R.; Bianchi, M.E. High Mobility Group B2 is Secreted by Myeloid Cells and has Mitogenic and Chemoattractant Activities Similar to High Mobility Group B1. *Autoimmunity* **2009**, *42*, 308–310.
3. Ugrinova, I.; Pashev, I.G.; Pasheva, E.A. Nucleosome Binding Properties and Co-Remodeling Activities of Native and in Vivo Acetylated HMGB-1 and HMGB-2 Proteins. *Biochemistry* **2009**, *48*, 6502–6507.
4. Swanson, P.C. Fine Structure and Activity of Discrete RAG-HMG Complexes on V(D)J Recombination Signals. *Mol. Cell. Biol.* **2002**, *22*, 1340–1351.
5. Bagherpoor, A.J.; Dolezalova, D.; Barta, T.; Kucirek, M.; Sani, S.A.; Esner, M.; Kunova Bosakova, M.; Vinarsky, V.; Peskova, L.; Hampl, A.; et al. Properties of Human Embryonic Stem Cells and their Differentiated Derivatives Depend on Nonhistone DNA-Binding HMGB1 and HMGB2 Proteins. *Stem Cells Dev.* **2017**, *26*, 328–340.
6. Ke, S.; Zhou, F.; Yang, H.; Wei, Y.; Gong, J.; Mei, Z.; Wu, L.; Yu, H.; Zhou, Y. Downregulation of High Mobility Group Box 1 Modulates Telomere Homeostasis and Increases the Radiosensitivity of Human Breast Cancer Cells. *Int. J. Oncol.* **2015**, *46*, 1051–1058.
7. Tang, D.; Kang, R.; Zeh, H.J., 3rd; Lotze, M.T. High-Mobility Group Box 1 and Cancer. *Biochim. Biophys. Acta* **2010**, *1799*, 131–140.
8. Liu, K.; Huang, J.; Xie, M.; Yu, Y.; Zhu, S.; Kang, R.; Cao, L.; Tang, D.; Duan, X. MIR34A Regulates Autophagy and Apoptosis by Targeting HMGB1 in the Retinoblastoma Cell. *Autophagy* **2014**, *10*, 442–452.
9. Chandrasekaran, K.S.; Sathyanarayanan, A.; Karunagaran, D. Downregulation of HMGB1 by miR-34a is Sufficient to Suppress Proliferation, Migration and Invasion of Human Cervical and Colorectal Cancer Cells. *Tumor Biol.* **2016**, *37*, 13155–13166.
10. Tang, C.; Yang, Z.; Chen, D.; Xie, Q.; Peng, T.; Wu, J.; Qi, S. Downregulation of miR-130a Promotes Cell Growth and Epithelial to Mesenchymal Transition by Activating HMGB2 in Glioma. *Int. J. Biochem. Cell Biol.* **2017**, *93*, 25–31.
11. Liu, P.L.; Liu, W.L.; Chang, J.M.; Chen, Y.H.; Liu, Y.P.; Kuo, H.F.; Hsieh, C.C.; Ding, Y.S.; Chen, W.W.; Chong, I.W. MicroRNA-200c Inhibits Epithelial-Mesenchymal Transition, Invasion, and Migration of Lung Cancer by Targeting HMGB1. *PLoS ONE* **2017**, *12*, e0180844.
12. Zhang, J.; Shao, S.; Han, D.; Xu, Y.; Jiao, D.; Wu, J.; Yang, F.; Ge, Y.; Shi, S.; Li, Y.; et al. High Mobility Group Box 1 Promotes the Epithelial-to-Mesenchymal Transition in Prostate Cancer PC3 Cells Via the RAGE/NF-kappaB Signaling Pathway. *Int. J. Oncol.* **2018**, *53*, 659–671.
13. van Beijnum, J.R.; Nowak-Sliwinska, P.; van den Boezem, E.; Hautvast, P.; Buurman, W.A.; Griffioen, A.W. Tumor Angiogenesis is Enforced by Autocrine Regulation of High-Mobility Group Box 1. *Oncogene* **2013**, *32*, 363–374.
14. Wu, Z.B.; Cai, L.; Lin, S.J.; Xiong, Z.K.; Lu, J.L.; Mao, Y.; Yao, Y.; Zhou, L.F. High-Mobility Group Box 2 is Associated with Prognosis of Glioblastoma by Promoting Cell Viability, Invasion, and Chemotherapeutic Resistance. *Neuro Oncol.* **2013**, *15*, 1264–1275.
15. Wang, W.; Jiang, H.; Zhu, H.; Zhang, H.; Gong, J.; Zhang, L.; Ding, Q. Overexpression of High Mobility Group Box 1 and 2 is Associated with the Progression and Angiogenesis of Human Bladder Carcinoma. *Oncol. Lett.* **2013**, *5*, 884–888.
16. Tai, S.; Sun, Y.; Squires, J.M.; Zhang, H.; Oh, W.K.; Liang, C.Z.; Huang, J. PC3 is a Cell Line Characteristic of Prostatic Small Cell Carcinoma. *Prostate* **2011**, *71*, 1668–1679.
17. Elangovan, I.; Thirugnanam, S.; Chen, A.; Zheng, G.; Bosland, M.C.; Kajdacsy-Balla, A.; Gnanasekar, M. Targeting Receptor for Advanced Glycation End Products (RAGE) Expression Induces Apoptosis and Inhibits Prostate Tumor Growth. *Biochem. Biophys. Res. Commun.* **2012**, *417*, 1133–1138.
18. Li, T.; Gui, Y.; Yuan, T.; Liao, G.; Bian, C.; Jiang, Q.; Huang, S.; Liu, B.; Wu, D. Overexpression of High Mobility Group Box 1 with Poor Prognosis in Patients After Radical Prostatectomy. *BJU Int.* **2012**, *110*, E1125–E1130.

19. Gnanasekar, M.; Thirugnanam, S.; Ramaswamy, K. Short Hairpin RNA (shRNA) Constructs Targeting High Mobility Group Box-1 (HMGB1) Expression Leads to Inhibition of Prostate Cancer Cell Survival and Apoptosis. *Int. J. Oncol.* **2009**, *34*, 425–431.
20. Flores-Morales, A.; Iglesias-Gato, D. Quantitative Mass Spectrometry-Based Proteomic Profiling for Precision Medicine in Prostate Cancer. *Front. Oncol.* **2017**, *7*, e267.
21. Muller, A.K.; Foll, M.; Heckelmann, B.; Kiefer, S.; Werner, M.; Schilling, O.; Biniossek, M.L.; Jilg, C.A.; Drendel, V. Proteomic Characterization of Prostate Cancer to Distinguish Nonmetastasizing and Metastasizing Primary Tumors and Lymph Node Metastases. *Neoplasia* **2018**, *20*, 140–151.
22. Zhang, Y.; Wang, D.; Li, M.; Wei, X.; Liu, S.; Zhao, M.; Liu, C.; Wang, X.; Jiang, X.; Li, X.; et al. Quantitative Proteomics of TRAMP Mice Combined with Bioinformatics Analysis Reveals that PDGF-B Regulatory Network Plays a Key Role in Prostate Cancer Progression. *J. Proteome Res.* **2018**, *17*, 2401–2411.
23. Stelloo, S.; Nevedomskaya, E.; Kim, Y.; Hoekman, L.; Bleijerveld, O.B.; Mirza, T.; Wessels, L.F.A.; van Weerden, W.M.; Altelaar, A.F.M.; Bergman, A.M.; et al. Endogenous Androgen Receptor Proteomic Profiling Reveals Genomic Subcomplex Involved in Prostate Tumorigenesis. *Oncogene* **2018**, *37*, 313–322.
24. Berger, A.; Brady, N.J.; Bareja, R.; Robinson, B.D.; Conteduca, V.; Augello, M.A.; Puca, L.; Ahmed, A.; Dardenne, E.; Lu, X.; et al. N-Myc-Mediated Epigenetic Reprogramming Drives Lineage Plasticity in Advanced Prostate Cancer. *J. Clin. Investig.* **2019**, *130*, 3924–3940.
25. Zhang, Z.; Chng, K.R.; Lingadahalli, S.; Chen, Z.; Liu, M.H.; Do, H.H.; Cai, S.; Rinaldi, N.; Poh, H.M.; Li, G.; et al. An AR-ERG Transcriptional Signature Defined by Long-Range Chromatin Interactomes in Prostate Cancer Cells. *Genome Res.* **2019**, *29*, 223–235.
26. Barreiro-Alonso, A.; Lamas-Maceiras, M.; Cerdan, E.M.; Vizoso-Vazquez, A. The HMGB Protein Ixr1 Interacts with Ssn8 and Tdh3 Involved in Transcriptional Regulation. *FEMS Yeast Res.* **2018**, *18*, doi:10.1093/femsyr/foy013.
27. Cerami, E.; Gao, J.; Dogrusoz, U.; Gross, B.E.; Sumer, S.O.; Aksoy, B.A.; Jacobsen, A.; Byrne, C.J.; Heuer, M.L.; Larsson, E.; et al. The cBio Cancer Genomics Portal: An Open Platform for Exploring Multidimensional Cancer Genomics Data. *Cancer Discov.* **2012**, *2*, 401–404.
28. Gao, J.; Aksoy, B.A.; Dogrusoz, U.; Dresdner, G.; Gross, B.; Sumer, S.O.; Sun, Y.; Jacobsen, A.; Sinha, R.; Larsson, E.; et al. Integrative Analysis of Complex Cancer Genomics and Clinical Profiles using the cBioPortal. *Sci. Signal.* **2013**, *6*, pl1.
29. Baca, S.C.; Prandi, D.; Lawrence, M.S.; Mosquera, J.M.; Romanel, A.; Drier, Y.; Park, K.; Kitabayashi, N.; MacDonald, T.Y.; Ghandi, M.; et al. Punctuated Evolution of Prostate Cancer Genomes. *Cell* **2013**, *153*, 666–677.
30. Barbieri, C.E.; Baca, S.C.; Lawrence, M.S.; Demichelis, F.; Blattner, M.; Theurillat, J.P.; White, T.A.; Stojanov, P.; Van Allen, E.; Stransky, N.; et al. Exome Sequencing Identifies Recurrent SPOP, FOXA1 and MED12 Mutations in Prostate Cancer. *Nat. Genet.* **2012**, *44*, 685–689.
31. Fraser, M.; Sabelnykova, V.Y.; Yamaguchi, T.N.; Heisler, L.E.; Livingstone, J.; Huang, V.; Shiah, Y.J.; Yousif, F.; Lin, X.; Masella, A.P.; et al. Genomic Hallmarks of Localized, Non-Indolent Prostate Cancer. *Nature* **2017**, *541*, 359–364.
32. Leyh-Bannurah, S.R.; Gazdovich, S.; Budaus, L.; Zaffuto, E.; Briganti, A.; Abdollah, F.; Montorsi, F.; Schiffmann, J.; Menon, M.; Shariat, S.F.; et al. Local Therapy Improves Survival in Metastatic Prostate Cancer. *Eur. Urol.* **2017**, *72*, 118–124.
33. Kumar, A.; Coleman, I.; Morrissey, C.; Zhang, X.; True, L.D.; Gulati, R.; Etzioni, R.; Bolouri, H.; Montgomery, B.; White, T.; et al. Substantial Interindividual and Limited Intraindividual Genomic Diversity among Tumors from Men with Metastatic Prostate Cancer. *Nat. Med.* **2016**, *22*, 369–378.
34. Taylor, B.S.; Schultz, N.; Hieronymus, H.; Gopalan, A.; Xiao, Y.; Carver, B.S.; Arora, V.K.; Kaushik, P.; Cerami, E.; Reva, B.; et al. Integrative Genomic Profiling of Human Prostate Cancer. *Cancer Cell* **2010**, *18*, 11–22.
35. Armenia, J.; Wankowicz, S.A.M.; Liu, D.; Gao, J.; Kundra, R.; Reznik, E.; Chatila, W.K.; Chakravarty, D.; Han, G.C.; Coleman, I.; et al. The Long Tail of Oncogenic Drivers in Prostate Cancer. *Nat. Genet.* **2018**, *50*, 645–651.
36. Rosario, S.R.; Long, M.D.; Affronti, H.C.; Rowsam, A.M.; Eng, K.H.; Smiraglia, D.J. Pan-Cancer Analysis of Transcriptional Metabolic Dysregulation using the Cancer Genome Atlas. *Nat. Commun.* **2018**, *9*, e5330.

37. Hieronymus, H.; Schultz, N.; Gopalan, A.; Carver, B.S.; Chang, M.T.; Xiao, Y.; Heguy, A.; Huberman, K.; Bernstein, M.; Assel, M.; et al. Copy Number Alteration Burden Predicts Prostate Cancer Relapse. *Proc. Natl. Acad. Sci. USA* **2014**, *111*, 11139–11144.
38. Gao, D.; Vela, I.; Sboner, A.; Iaquinta, P.J.; Karthaus, W.R.; Gopalan, A.; Dowling, C.; Wanjala, J.N.; Undvall, E.A.; Arora, V.K.; et al. Organoid Cultures Derived from Patients with Advanced Prostate Cancer. *Cell* **2014**, *159*, 176–187.
39. Abida, W.; Armenia, J.; Gopalan, A.; Brennan, R.; Walsh, M.; Barron, D.; Danila, D.; Rathkopf, D.; Morris, M.; Slovin, S.; et al. Prospective Genomic Profiling of Prostate Cancer Across Disease States Reveals Germline and Somatic Alterations that may Affect Clinical Decision Making. *JCO Precis. Oncol.* **2017**, doi:10.1200/PO.17.00029.
40. Grasso, C.S.; Wu, Y.M.; Robinson, D.R.; Cao, X.; Dhanasekaran, S.M.; Khan, A.P.; Quist, M.J.; Jing, X.; Lonigro, R.J.; Brenner, J.C.; et al. The Mutational Landscape of Lethal Castration-Resistant Prostate Cancer. *Nature* **2012**, *487*, 239–243.
41. Abida, W.; Cyrta, J.; Heller, G.; Prandi, D.; Armenia, J.; Coleman, I.; Cieslik, M.; Benelli, M.; Robinson, D.; Van Allen, E.M.; et al. Genomic Correlates of Clinical Outcome in Advanced Prostate Cancer. *Proc. Natl. Acad. Sci. USA* **2019**, *116*, 11428–11436.
42. Robinson, D.; Van Allen, E.M.; Wu, Y.M.; Schultz, N.; Lonigro, R.J.; Mosquera, J.M.; Montgomery, B.; Taplin, M.E.; Pritchard, C.C.; Attard, G.; et al. Integrative Clinical Genomics of Advanced Prostate Cancer. *Cell* **2015**, *161*, 1215–1228.
43. Beltran, H.; Prandi, D.; Mosquera, J.M.; Benelli, M.; Puca, L.; Cyrta, J.; Marotz, C.; Giannopoulou, E.; Chakravarthi, B.V.; Varambally, S.; et al. Divergent Clonal Evolution of Castration-Resistant Neuroendocrine Prostate Cancer. *Nat. Med.* **2016**, *22*, 298–305.
44. Liu, R.; Zhou, Z.; Huang, J.; Chen, C. PMEPA1 Promotes Androgen Receptor-Negative Prostate Cell Proliferation through Suppressing the Smad3/4-c-Myc-p21 Cip1 Signaling Pathway. *J. Pathol.* **2011**, *223*, 683–694.
45. Zhao, C.B.; Bao, J.M.; Lu, Y.J.; Zhao, T.; Zhou, X.H.; Zheng, D.Y.; Zhao, S.C. Co-Expression of RAGE and HMGB1 is Associated with Cancer Progression and Poor Patient Outcome of Prostate Cancer. *Am. J. Cancer Res.* **2014**, *4*, 369–377.
46. Bull, J.H.; Ellison, G.; Patel, A.; Muir, G.; Walker, M.; Underwood, M.; Khan, F.; Paskins, L. Identification of Potential Diagnostic Markers of Prostate Cancer and Prostatic Intraepithelial Neoplasia using cDNA Microarray. *Br. J. Cancer* **2001**, *84*, 1512–1519.
47. Daures, M.; Idrissou, M.; Judes, G.; Rifai, K.; Penault-Llorca, F.; Bignon, Y.J.; Guy, L.; Bernard-Gallon, D. A New Metabolic Gene Signature in Prostate Cancer Regulated by JMJD3 and EZH2. *Oncotarget* **2018**, *9*, 23413–23425.
48. Amamoto, R.; Yagi, M.; Song, Y.; Oda, Y.; Tsuneyoshi, M.; Naito, S.; Yokomizo, A.; Kuroiwa, K.; Tokunaga, S.; Kato, S.; et al. Mitochondrial p32/C1QBP is Highly Expressed in Prostate Cancer and is Associated with Shorter Prostate-Specific Antigen Relapse Time After Radical Prostatectomy. *Cancer Sci.* **2011**, *102*, 639–647.
49. Iglesias-Gato, D.; Wikstrom, P.; Tyanova, S.; Lavallee, C.; Thysell, E.; Carlsson, J.; Hagglof, C.; Cox, J.; Andren, O.; Stattin, P.; et al. The Proteome of Primary Prostate Cancer. *Eur. Urol.* **2016**, *69*, 942–952.
50. Rajan, P.; Sudbery, I.M.; Villasevil, M.E.; Mui, E.; Fleming, J.; Davis, M.; Ahmad, I.; Edwards, J.; Sansom, O.J.; Sims, D.; et al. Next-Generation Sequencing of Advanced Prostate Cancer Treated with Androgen-Deprivation Therapy. *Eur. Urol.* **2014**, *66*, 32–39.
51. Kim, J.Y.; Cho, Y.E.; Kim, G.Y.; Lee, H.L.; Lee, S.; Park, J.H. Down-Regulation and Aberrant Cytoplasmic Expression of GLTSCR2 in Prostatic Adenocarcinomas. *Cancer Lett.* **2013**, *340*, 134–140.
52. Kashyap, V.; Bonavida, B. Role of YY1 in the Pathogenesis of Prostate Cancer and Correlation with Bioinformatic Data Sets of Gene Expression. *Genes Cancer* **2014**, *5*, 71–83.
53. Park, A.; Lee, J.; Mun, S.; Kim, D.J.; Cha, B.H.; Moon, K.T.; Yoo, T.K.; Kang, H.G. Identification of Transcription Factor YY1 as a Regulator of a Prostate Cancer-Specific Pathway using Proteomic Analysis. *J. Cancer* **2017**, *8*, 2303–2311.
54. Huang, Y.; Tao, T.; Liu, C.; Guan, H.; Zhang, G.; Ling, Z.; Zhang, L.; Lu, K.; Chen, S.; Xu, B.; et al. Upregulation of miR-146a by YY1 Depletion Correlates with Delayed Progression of Prostate Cancer. *Int. J. Oncol.* **2017**, *50*, 421–431.

55. Yang, T.; An, Z.; Zhang, C.; Wang, Z.; Wang, X.; Liu, Y.; Du, E.; Liu, R.; Zhang, Z.; Xu, Y. HnRNPM, a Potential Mediator of YY1 in Promoting the Epithelial-Mesenchymal Transition of Prostate Cancer Cells. *Prostate* **2019**, *79*, 1199–1210.
56. Camacho-Moctezuma, B.; Quevedo-Castillo, M.; Melendez-Zajgla, J.; Aquino-Jarquin, G.; Martinez-Ruiz, G.U. YY1 Negatively Regulates the XAF1 Gene Expression in Prostate Cancer. *Biochem. Biophys. Res. Commun.* **2019**, *508*, 973–979.
57. Deng, Z.; Cao, P.; Wan, M.M.; Sui, G. Yin Yang 1: A Multifaceted Protein Beyond a Transcription Factor. *Transcription* **2010**, *1*, 81–84.
58. Li, B.; Cao, X.; Weng, C.; Wu, Y.; Fang, X.; Zhang, X.; Liu, G. HoxA10 Induces Proliferation in Human Prostate Carcinoma PC-3 Cell Line. *Cell Biochem. Biophys.* **2014**, *70*, 1363–1368.
59. Hatanaka, Y.; de Velasco, M.A.; Oki, T.; Shimizu, N.; Nozawa, M.; Yoshimura, K.; Yoshikawa, K.; Nishio, K.; Uemura, H. HOXA10 Expression Profiling in Prostate Cancer. *Prostate* **2019**, *79*, 554–563.
60. Long, Z.; Li, Y.; Gan, Y.; Zhao, D.; Wang, G.; Xie, N.; Lovnicki, J.M.; Fazli, L.; Cao, Q.; Chen, K.; et al. Roles of the HOXA10 Gene during Castrate-Resistant Prostate Cancer Progression. *Endocr. Relat. Cancer* **2019**, *26*, 279–292.
61. GTEx Consortium. The Genotype-Tissue Expression (GTEx) Project. *Nat. Genet.* **2013**, *45*, 580–585.
62. Ravipaty, S.; Wu, W.; Dalvi, A.; Tanna, N.; Andreazi, J.; Friss, T.; Klotz, A.; Liao, C.; Garren, J.; Schofield, S.; et al. Clinical Validation of a Serum Protein Panel (FLNA, FLNB and KRT19) for Diagnosis of Prostate Cancer. *J. Mol. Biomark. Diagn.* **2017**, *8*, doi:10.4172/2155-9929.1000323.
63. Romanuik, T.L.; Ueda, T.; Le, N.; Haile, S.; Yong, T.M.; Thomson, T.; Vessella, R.L.; Sadar, M.D. Novel Biomarkers for Prostate Cancer Including Noncoding Transcripts. *Am. J. Pathol.* **2009**, *175*, 2264–2276.
64. Thomas, B.C.; Kay, J.D.; Menon, S.; Vowler, S.L.; Dawson, S.N.; Bucklow, L.J.; Luxton, H.J.; Johnston, T.; Massie, C.E.; Pugh, M.; et al. Whole Blood mRNA in Prostate Cancer Reveals a Four-Gene Androgen Regulated Panel. *Endocr. Relat. Cancer* **2016**, *23*, 797–812.
65. Kaighn, M.E.; Narayan, K.S.; Ohnuki, Y.; Lechner, J.F.; Jones, L.W. Establishment and Characterization of a Human Prostatic Carcinoma Cell Line (PC-3). *Investig. Urol.* **1979**, *17*, 16–23.
66. Livak, K.J.; Schmittgen, T.D. Analysis of Relative Gene Expression Data using Real-Time Quantitative PCR and the 2(-Delta Delta C(T)) Method. *Methods* **2001**, *25*, 402–408.
67. Babicki, S.; Arndt, D.; Marcu, A.; Liang, Y.; Grant, J.R.; Maciejewski, A.; Wishart, D.S. Heatmapper: Web-Enabled Heat Mapping for all. *Nucleic Acids Res.* **2016**, *44*, W147–W153.
68. Petryszak, R.; Keays, M.; Tang, Y.A.; Fonseca, N.A.; Barrera, E.; Burdett, T.; Fullgrabe, A.; Fuentes, A.M.; Jupp, S.; Koskinen, S.; et al. Expression Atlas Update, an Integrated Database of Gene and Protein Expression in Humans, Animals and Plants. *Nucleic Acids Res.* **2016**, *44*, D746–D752.
69. Chen, R.; Xiao, M.; Gao, H.; Chen, Y.; Li, Y.; Liu, Y.; Zhang, N. Identification of a Novel Mitochondrial Interacting Protein of C1QBP using Subcellular Fractionation Coupled with CoIP-MS. *Anal. Bioanal. Chem.* **2016**, *408*, 1557–1564.
70. Li, C.; He, C.; Xu, Y.; Xu, H.; Tang, Y.; Chavan, H.; Duan, S.; Artigues, A.; Forrest, M.L.; Krishnamurthy, P.; et al. Alternol Eliminates Excessive ATP Production by Disturbing Krebs Cycle in Prostate Cancer. *Prostate* **2019**, *79*, 628–639.
71. Omran, H.; Kobayashi, D.; Olbrich, H.; Tsukahara, T.; Loges, N.T.; Hagiwara, H.; Zhang, Q.; Leblond, G.; O'Toole, E.; Hara, C.; et al. Ktu/PF13 is Required for Cytoplasmic Pre-Assembly of Axonemal Dyneins. *Nature* **2008**, *456*, 611–616.
72. Massafra, V.; Milona, A.; Vos, H.R.; Burgering, B.M.; van Mil, S.W. Quantitative Liver Proteomics Identifies FGF19 Targets that Couple Metabolism and Proliferation. *PLoS ONE* **2017**, *12*, e0171185.
73. Zhou, S.; Lu, J.; Li, Y.; Chen, C.; Cai, Y.; Tan, G.; Peng, Z.; Zhang, Z.; Dong, Z.; Kang, T.; et al. MNAT1 is Overexpressed in Colorectal Cancer and Mediates p53 Ubiquitin-Degradation to Promote Colorectal Cancer Malignance. *J. Exp. Clin. Cancer Res.* **2018**, *37*, e284.
74. Wang, W.; Chen, Z.; Mao, Z.; Zhang, H.; Ding, X.; Chen, S.; Zhang, X.; Xu, R.; Zhu, B. Nucleolar Protein Spindlin1 Recognizes H3K4 Methylation and Stimulates the Expression of rRNA Genes. *EMBO Rep.* **2011**, *12*, 1160–1166.
75. Plafker, K.S.; Plafker, S.M. The Ubiquitin-Conjugating Enzyme UBE2E3 and its Import Receptor Importin-11 Regulate the Localization and Activity of the Antioxidant Transcription Factor NRF2. *Mol. Biol. Cell* **2015**, *26*, 327–338.

76. Huang, K.; Yang, C.; Wang, Q.X.; Li, Y.S.; Fang, C.; Tan, Y.L.; Wei, J.W.; Wang, Y.F.; Li, X.; Zhou, J.H.; et al. The CRISPR/Cas9 System Targeting EGFR Exon 17 Abrogates NF-kappaB Activation Via Epigenetic Modulation of UBXN1 in EGFRwt/vIII Glioma Cells. *Cancer Lett.* **2017**, *388*, 269–280.
77. Lu, H.; Hallstrom, T.C. The Nuclear Protein UHRF2 is a Direct Target of the Transcription Factor E2F1 in the Induction of Apoptosis. *J. Biol. Chem.* **2013**, *288*, 23833–23843.
78. McGee, A.M.; Douglas, D.L.; Liang, Y.; Hyder, S.M.; Baines, C.P. The Mitochondrial Protein C1qbp Promotes Cell Proliferation, Migration and Resistance to Cell Death. *Cell Cycle* **2011**, *10*, 4119–4127.
79. Quintero, I.B.; Herrala, A.M.; Araujo, C.L.; Pulkka, A.E.; Hautaniemi, S.; Ovaska, K.; Pryazhnikov, E.; Kulesskiy, E.; Ruuth, M.K.; Soini, Y.; et al. Transmembrane Prostatic Acid Phosphatase (TMPAP) Interacts with Snapin and Deficient Mice Develop Prostate Adenocarcinoma. *PLoS ONE* **2013**, 8, e73072.
80. Fei, D.L.; Motowski, H.; Chatrikhi, R.; Prasad, S.; Yu, J.; Gao, S.; Kielkopf, C.L.; Bradley, R.K.; Varmus, H. Wild-Type U2AF1 Antagonizes the Splicing Program Characteristic of U2AF1-Mutant Tumors and is Required for Cell Survival. *PLoS Genet.* **2016**, *12*, e1006384.
81. Shankar, E.; Song, K.; Corum, S.L.; Bane, K.L.; Wang, H.; Kao, H.Y.; Danielpour, D. A Signaling Network Controlling Androgenic Repression of c-Fos Protein in Prostate Adenocarcinoma Cells. *J. Biol. Chem.* **2016**, *291*, 5512–5526.
82. Yan, G.; Ru, Y.; Wu, K.; Yan, F.; Wang, Q.; Wang, J.; Pan, T.; Zhang, M.; Han, H.; Li, X.; et al. GOLM1 Promotes Prostate Cancer Progression through Activating PI3K-AKT-mTOR Signaling. *Prostate* **2018**, *78*, 166–177.
83. Zhang, L.; Song, D.; Zhu, B.; Wang, X. The Role of Nuclear Matrix Protein HNRNPU in Maintaining the Architecture of 3D Genome. *Semin. Cell Dev. Biol.* **2019**, *90*, 161–167.
84. Lee, S.Y.; Kim, J.W.; Jeong, M.H.; An, J.H.; Jang, S.M.; Song, K.H.; Choi, K.H. Microtubule-Associated Protein 1B Light Chain (MAP1B-LC1) Negatively Regulates the Activity of Tumor Suppressor p53 in Neuroblastoma Cells. *FEBS Lett.* **2008**, *582*, 2826–2832.
85. Zheng, M.; Wang, Y.H.; Wu, X.N.; Wu, S.Q.; Lu, B.J.; Dong, M.Q.; Zhang, H.; Sun, P.; Lin, S.C.; Guan, K.L.; et al. Inactivation of Rheb by PRAK-Mediated Phosphorylation is Essential for Energy-Depletion-Induced Suppression of mTORC1. *Nat. Cell Biol.* **2011**, *13*, 263–272.
86. Dwyer, S.F.; Gelman, I.H. Cross-Phosphorylation and Interaction between Src/FAK and MAPKAP5/PRAK in Early Focal Adhesions Controls Cell Motility. *J. Cancer Biol. Res.* **2014**, *2*, e1045.
87. Dasgupta, S.; Wasson, L.M.; Rauniyar, N.; Prokai, L.; Borejdo, J.; Vishwanatha, J.K. Novel Gene C17orf37 in 17q12 Amplicon Promotes Migration and Invasion of Prostate Cancer Cells. *Oncogene* **2009**, *28*, 2860–2872.
88. Chen, S.H.; Chen, L.; Russell, D.H. Metal-Induced Conformational Changes of Human Metallothionein-2A: A Combined Theoretical and Experimental Study of Metal-Free and Partially Metalated Intermediates. *J. Am. Chem. Soc.* **2014**, *136*, 9499–9508.
89. Yamasaki, M.; Nomura, T.; Sato, F.; Mimata, H. Metallothionein is Up-Regulated Under Hypoxia and Promotes the Survival of Human Prostate Cancer Cells. *Oncol. Rep.* **2007**, *18*, 1145–1153.
90. Lin, H.K.; Altuwaijri, S.; Lin, W.J.; Kan, P.Y.; Collins, L.L.; Chang, C. Proteasome Activity is Required for Androgen Receptor Transcriptional Activity Via Regulation of Androgen Receptor Nuclear Translocation and Interaction with Coregulators in Prostate Cancer Cells. *J. Biol. Chem.* **2002**, *277*, 36570–36576.
91. Cho, S.; Choi, Y.J.; Kim, J.M.; Jeong, S.T.; Kim, J.H.; Kim, S.H.; Ryu, S.E. Binding and Regulation of HIF-1alpha by a Subunit of the Proteasome Complex, PSMA7. *FEBS Lett.* **2001**, *498*, 62–66.
92. Kim, M.; Morales, L.D.; Jang, I.S.; Cho, Y.Y.; Kim, D.J. Protein Tyrosine Phosphatases as Potential Regulators of STAT3 Signaling. *Int. J. Mol. Sci.* **2018**, *19*, e2708.
93. Hui, K.; Gao, Y.; Huang, J.; Xu, S.; Wang, B.; Zeng, J.; Fan, J.; Wang, X.; Yue, Y.; Wu, S.; et al. RASAL2, a RAS GTPase-Activating Protein, Inhibits Stemness and Epithelial-Mesenchymal Transition Via MAPK/SOX2 Pathway in Bladder Cancer. *Cell Death Dis.* **2017**, *8*, e2600.
94. Min, S.; Kim, K.; Kim, S.G.; Cho, H.; Lee, Y. Chromatin-Remodeling Factor, RSF1, Controls p53-Mediated Transcription in Apoptosis upon DNA Strand Breaks. *Cell Death Dis.* **2018**, *9*, e1079.
95. Li, H.; Zhang, Y.; Zhang, Y.; Bai, X.; Peng, Y.; He, P. Rsf-1 Overexpression in Human Prostate Cancer, Implication as a Prognostic Marker. *Tumor Biol.* **2014**, *35*, 5771–5776.
96. Jia, R.; Ajiro, M.; Yu, L.; McCoy, P., Jr.; Zheng, Z.M. Oncogenic Splicing Factor SRSF3 Regulates ILF3 Alternative Splicing to Promote Cancer Cell Proliferation and Transformation. *RNA* **2019**, *25*, 630–644.

97. Bowler, E.; Porazinski, S.; Uzor, S.; Thibault, P.; Durand, M.; Lapointe, E.; Rouschop, K.M.A.; Hancock, J.; Wilson, I.; Ladomery, M. Hypoxia Leads to Significant Changes in Alternative Splicing and Elevated Expression of CLK Splice Factor Kinases in PC3 Prostate Cancer Cells. *BMC Cancer* **2018**, *18*, e355.
98. Bereczki, O.; Ujfaludi, Z.; Pardi, N.; Nagy, Z.; Tora, L.; Boros, I.M.; Balint, E. TATA Binding Protein Associated Factor 3 (TAF3) Interacts with p53 and Inhibits its Function. *BMC Mol. Biol.* **2008**, *9*, e57.
99. Fesus, L.; Thomazy, V.; Falus, A. Induction and Activation of Tissue Transglutaminase during Programmed Cell Death. *FEBS Lett.* **1987**, *224*, 104–108.
100. Moniz, S.; Jordan, P. Emerging Roles for WNK Kinases in Cancer. *Cell Mol. Life Sci.* **2010**, *67*, 1265–1276.
101. Seligson, D.; Horvath, S.; Huerta-Yepez, S.; Hanna, S.; Garban, H.; Roberts, A.; Shi, T.; Liu, X.; Chia, D.; Goodglick, L.; et al. Expression of Transcription Factor Yin Yang 1 in Prostate Cancer. *Int. J. Oncol.* **2005**, *27*, 131–141.
102. Riera-Romo, M. COMMD1: A Multifunctional Regulatory Protein. *J. Cell. Biochem.* **2018**, *119*, 34–51.
103. Zoubeidi, A.; Ettinger, S.; Beraldi, E.; Hadaschik, B.; Zardan, A.; Klomp, L.W.; Nelson, C.C.; Rennie, P.S.; Gleave, M.E. Clusterin Facilitates COMMD1 and I-kappaB Degradation to Enhance NF-kappaB Activity in Prostate Cancer Cells. *Mol. Cancer. Res.* **2010**, *8*, 119–130.
104. Loy, C.J.; Sim, K.S.; Yong, E.L. Filamin-A Fragment Localizes to the Nucleus to Regulate Androgen Receptor and Coactivator Functions. *Proc. Natl. Acad. Sci. USA* **2003**, *100*, 4562–4567.
105. Yeh, D.W.; Chen, Y.S.; Lai, C.Y.; Liu, Y.L.; Lu, C.H.; Lo, J.F.; Chen, L.; Hsu, L.C.; Luo, Y.; Xiang, R.; et al. Downregulation of COMMD1 by miR-205 Promotes a Positive Feedback Loop for Amplifying Inflammatory- and Stemness-Associated Properties of Cancer Cells. *Cell Death Differ.* **2016**, *23*, 841–852.
106. Park, I.; Han, C.; Jin, S.; Lee, B.; Choi, H.; Kwon, J.T.; Kim, D.; Kim, J.; Lifirsu, E.; Park, W.J.; et al. Myosin Regulatory Light Chains are Required to Maintain the Stability of Myosin II and Cellular Integrity. *Biochem. J.* **2011**, *434*, 171–180.
107. Lee, S.; Kim, J.Y.; Kim, Y.J.; Seok, K.O.; Kim, J.H.; Chang, Y.J.; Kang, H.Y.; Park, J.H. Nucleolar Protein GLTSCR2 Stabilizes p53 in Response to Ribosomal Stresses. *Cell Death Differ.* **2012**, *19*, 1613–1622.
108. Kim, H.K.; Fuchs, G.; Wang, S.; Wei, W.; Zhang, Y.; Park, H.; Roy-Chaudhuri, B.; Li, P.; Xu, J.; Chu, K.; et al. A Transfer-RNA-Derived Small RNA Regulates Ribosome Biogenesis. *Nature* **2017**, *552*, 57–62.
109. Gylfe, A.E.; Kondelin, J.; Turunen, M.; Ristolainen, H.; Katainen, R.; Pitkanen, E.; Kaasinen, E.; Rantanen, V.; Tanskanen, T.; Varjosalo, M.; et al. Identification of Candidate Oncogenes in Human Colorectal Cancers with Microsatellite Instability. *Gastroenterology* **2013**, *145*, 540–543.
110. Kim, J.Y.; An, Y.M.; Park, J.H. Role of GLTSCR2 in the Regulation of Telomerase Activity and Chromosome Stability. *Mol. Med. Rep.* **2016**, *14*, 1697–1703.
111. Sheu, J.J.; Guan, B.; Choi, J.H.; Lin, A.; Lee, C.H.; Hsiao, Y.T.; Wang, T.L.; Tsai, F.J.; Shih, I. Rsf-1, a Chromatin Remodeling Protein, Induces DNA Damage and Promotes Genomic Instability. *J. Biol. Chem.* **2010**, *285*, 38260–38269.
112. He, X.; Zhang, P. Serine/arginine-Rich Splicing Factor 3 (SRSF3) Regulates Homologous Recombination-Mediated DNA Repair. *Mol. Cancer* **2015**, *14*, e158.
113. Scully, O.J.; Yu, Y.; Salim, A.; Thike, A.A.; Yip, G.W.; Baeg, G.H.; Tan, P.H.; Matsumoto, K.; Bay, B.H. Complement Component 1, q Subcomponent Binding Protein is a Marker for Proliferation in Breast Cancer. *Exp. Biol. Med.* **2015**, *240*, 846–853.
114. Long, Y.; Wu, Z.; Yang, X.; Chen, L.; Han, Z.; Zhang, Y.; Liu, J.; Liu, W.; Liu, X. MicroRNA-101 Inhibits the Proliferation and Invasion of Bladder Cancer Cells Via Targeting c-FOS. *Mol. Med. Rep.* **2016**, *14*, 2651–2656.
115. Lu, C.; Shen, Q.; DuPre, E.; Kim, H.; Hilsenbeck, S.; Brown, P.H. CFos is Critical for MCF-7 Breast Cancer Cell Growth. *Oncogene* **2005**, *24*, 6516–6524.
116. Goh, W.Q.; Ow, G.S.; Kuznetsov, V.A.; Chong, S.; Lim, Y.P. DLAT Subunit of the Pyruvate Dehydrogenase Complex is Upregulated in Gastric Cancer-Implications in Cancer Therapy. *Am. J. Transl. Res.* **2015**, *7*, 1140–1151.
117. Salimi, R.; Bandaru, S.; Devarakonda, S.; Gokalp, S.; Ala, C.; Alvandian, A.; Yener, N.; Akyurek, L.M. Blocking the Cleavage of Filamin A by Calpain Inhibitor Decreases Tumor Cell Growth. *Anticancer Res.* **2018**, *38*, 2079–2085.
118. Gai, X.; Tang, B.; Liu, F.; Wu, Y.; Wang, F.; Jing, Y.; Huang, F.; Jin, D.; Wang, L.; Zhang, H. MTOR/miR-145-Regulated Exosomal GOLM1 Promotes Hepatocellular Carcinoma through Augmented GSK-3beta/MMPs. *J. Genet. Genom.* **2019**, *46*, 235–245.

119. Zhang, R.; Zhu, Z.; Shen, W.; Li, X.; Dhoomun, D.K.; Tian, Y. Golgi Membrane Protein 1 (GOLM1) Promotes Growth and Metastasis of Breast Cancer Cells Via Regulating Matrix Metalloproteinase-13 (MMP13). *Med. Sci. Monit.* **2019**, *25*, 847–855.
120. Yang, L.; Luo, P.; Song, Q.; Fei, X. DNMT1/miR-200a/GOLM1 Signaling Pathway Regulates Lung Adenocarcinoma Cells Proliferation. Biomed. *Pharmacother* **2018**, *99*, 839–847.
121. Li, B.; Jin, H.; Yu, Y.; Gu, C.; Zhou, X.; Zhao, N.; Feng, Y. HOXA10 is Overexpressed in Human Ovarian Clear Cell Adenocarcinoma and Correlates with Poor Survival. *Int. J. Gynecol. Cancer* **2009**, *19*, 1347–1352.
122. Tang, W.; Jiang, Y.; Mu, X.; Xu, L.; Cheng, W.; Wang, X. MiR-135a Functions as a Tumor Suppressor in Epithelial Ovarian Cancer and Regulates HOXA10 Expression. *Cell. Signal.* **2014**, *26*, 1420–1426.
123. Moon, A.; Lim, S.J.; Jo, Y.H.; Lee, S.; Kim, J.Y.; Lee, J.; Park, J.H. Downregulation of GLTSCR2 Expression is Correlated with Breast Cancer Progression. *Pathol. Res. Pract.* **2013**, *209*, 700–704.
124. Ren, C.C.; Yang, L.; Liu, L.; Chen, Y.N.; Cheng, G.M.; Zhang, X.A.; Liu, H. Effects of shRNA-Mediated Silencing of PSMA7 on Cell Proliferation and Vascular Endothelial Growth Factor Expression Via the Ubiquitin-Proteasome Pathway in Cervical Cancer. *J. Cell. Physiol.* **2019**, *234*, 5851–5862.
125. Morales, L.D.; Archbold, A.K.; Olivarez, S.; Slaga, T.J.; DiGiovanni, J.; Kim, D.J. The Role of T-Cell Protein Tyrosine Phosphatase in Epithelial Carcinogenesis. *Mol. Carcinog.* **2019**, *58*, 1640–1647.
126. Fang, J.F.; Zhao, H.P.; Wang, Z.F.; Zheng, S.S. Upregulation of RASAL2 Promotes Proliferation and Metastasis, and is Targeted by miR-203 in Hepatocellular Carcinoma. *Mol. Med. Rep.* **2017**, *15*, 2720–2726.
127. Liu, Y.; Li, G.; Liu, C.; Tang, Y.; Zhang, S. RSF1 Regulates the Proliferation and Paclitaxel Resistance Via Modulating NF-kappaB Signaling Pathway in Nasopharyngeal Carcinoma. *J. Cancer.* **2017**, *8*, 354–362.
128. Li, Y.; Ma, X.; Wang, Y.; Li, G. MiR-489 Inhibits Proliferation, Cell Cycle Progression and Induces Apoptosis of Glioma Cells Via Targeting SPIN1-Mediated PI3K/AKT Pathway. *Biomed. Pharmacother.* **2017**, *93*, 435–443.
129. Li, W.; Zhang, Z.; Zhao, W.; Han, N. Transglutaminase 3 Protein Modulates Human Esophageal Cancer Cell Growth by Targeting the NF-kappaB Signaling Pathway. *Oncol. Rep.* **2016**, *36*, 1723–1730.
130. Plafker, K.S.; Farjo, K.M.; Wiechmann, A.F.; Plafker, S.M. The Human Ubiquitin Conjugating Enzyme, UBE2E3, is Required for Proliferation of Retinal Pigment Epithelial Cells. *Investig. Ophthalmol. Vis. Sci.* **2008**, *49*, 5611–5618.
131. Yang, W.L.; Wei, L.; Huang, W.Q.; Li, R.; Shen, W.Y.; Liu, J.Y.; Xu, J.M.; Li, B.; Qin, Y. Vigilin is Overexpressed in Hepatocellular Carcinoma and is Required for HCC Cell Proliferation and Tumor Growth. *Oncol. Rep.* **2014**, *31*, 2328–2334.
132. Mu, P.; Akashi, T.; Lu, F.; Kishida, S.; Kadomatsu, K. A Novel Nuclear Complex of DRR1, F-Actin and COMMD1 Involved in NF-kappaB Degradation and Cell Growth Suppression in Neuroblastoma. *Oncogene* **2017**, *36*, 5745–5756.
133. Vitali, E.; Boemi, I.; Rosso, L.; Cambiaghi, V.; Novellis, P.; Mantovani, G.; Spada, A.; Alloisio, M.; Veronesi, G.; Ferrero, S.; et al. FLNA is Implicated in Pulmonary Neuroendocrine Tumors Aggressiveness and Progression. *Oncotarget* **2017**, *8*, 77330–77340.
134. Wei, L.; Xie, X.; Li, J.; Li, R.; Shen, W.; Duan, S.; Zhao, R.; Yang, W.; Liu, Q.; Fu, Q.; et al. Disruption of Human Vigilin Impairs Chromosome Condensation and Segregation. *Cell Biol. Int.* **2015**, *39*, 1234–1241.
135. Harrison, B.; Kraus, M.; Burch, L.; Stevens, C.; Craig, A.; Gordon-Weeks, P.; Hupp, T.R. DAPK-1 Binding to a Linear Peptide Motif in MAP1B Stimulates Autophagy and Membrane Blebbing. *J. Biol. Chem.* **2008**, *283*, 9999–10014.
136. Zhou, J.; Wan, B.; Liu, X.M.; Li, R.; Wang, Y.; Yu, L. MK5 is Degraded in Response to Doxorubicin and Negatively Regulates Doxorubicin-Induced Apoptosis in Hepatocellular Carcinoma Cells. *Biochem. Biophys. Res. Commun.* **2012**, *427*, 581–586.
137. Marikar, F.M.; Jin, G.; Sheng, W.; Ma, D.; Hua, Z. Metallothionein 2A an Interactive Protein Linking Phosphorylated FADD to NF-kappaB Pathway Leads to Colorectal Cancer Formation. *Chin. Clin. Oncol.* **2016**, *5*, e76.
138. Yang, L.; Tang, Z.; Zhang, H.; Kou, W.; Lu, Z.; Li, X.; Li, Q.; Miao, Z. PSMA7 Directly Interacts with NOD1 and Regulates its Function. *Cell. Physiol. Biochem.* **2013**, *31*, 952–959.
139. Zhou, L.; Guo, J.; Jia, R. Oncogene SRSF3 Suppresses Autophagy Via Inhibiting BECN1 Expression. *Biochem. Biophys. Res. Commun.* **2019**, *509*, 966–972.

140. Kim, J.; Park, R.Y.; Chen, J.K.; Kim, J.; Jeong, S.; Ohn, T. Splicing Factor SRSF3 Represses the Translation of Programmed Cell Death 4 mRNA by Associating with the 5'-UTR Region. *Cell Death Differ.* **2014**, *21*, 481–490.
141. He, X.; Arslan, A.D.; Pool, M.D.; Ho, T.T.; Darcy, K.M.; Coon, J.S.; Beck, W.T. Knockdown of Splicing Factor SRp20 Causes Apoptosis in Ovarian Cancer Cells and its Expression is Associated with Malignancy of Epithelial Ovarian Cancer. *Oncogene* **2011**, *30*, 356–365.
142. Galluzzi, L.; Bravo-San Pedro, J.M.; Kroemer, G. Defective Autophagy Initiates Malignant Transformation. *Mol. Cell* **2016**, *62*, 473–474.
143. Bonavida, B. Linking Autophagy and the Dysregulated NFkappaB/ SNAIL/YY1/RKIP/PTEN Loop in Cancer: Therapeutic Implications. *Crit. Rev. Oncog.* **2018**, *23*, 307–320.
144. Zhang, X.; Zhang, F.; Guo, L.; Wang, Y.; Zhang, P.; Wang, R.; Zhang, N.; Chen, R. Interactome Analysis Reveals that C1QBP (Complement Component 1, q Subcomponent Binding Protein) is Associated with Cancer Cell Chemotaxis and Metastasis. *Mol. Cell. Proteom.* **2013**, *12*, 3199–3209.
145. van de Sluis, B.; Mao, X.; Zhai, Y.; Groot, A.J.; Vermeulen, J.F.; van der Wall, E.; van Diest, P.J.; Hofker, M.H.; Wijmenga, C.; Klomp, L.W.; et al. COMMD1 Disrupts HIF-1alpha/beta Dimerization and Inhibits Human Tumor Cell Invasion. *J. Clin. Investig.* **2010**, *120*, 2119–2130.
146. Zhou, M.; Chen, X.; Wu, J.; He, X.; Ren, R. MicroRNA-143 Regulates Cell Migration and Invasion by Targeting GOLM1 in Cervical Cancer. *Oncol. Lett.* **2018**, *16*, 6393–6400.
147. Kpetemey, M.; Chaudhary, P.; Van Treuren, T.; Vishwanatha, J.K. MIEN1 Drives Breast Tumor Cell Migration by Regulating Cytoskeletal-Focal Adhesion Dynamics. *Oncotarget* **2016**, *7*, 54913–54924.
148. Rajendiran, S.; Parwani, A.V.; Hare, R.J.; Dasgupta, S.; Roby, R.K.; Vishwanatha, J.K. MicroRNA-940 Suppresses Prostate Cancer Migration and Invasion by Regulating MIEN1. *Mol. Cancer* **2014**, *13*, e250.
149. Yoshimoto, M.; Tokuda, A.; Nishiwaki, K.; Sengoku, K.; Yaginuma, Y. Abnormal Expression of PICT-1 and its Codon 389 Polymorphism is a Risk Factor for Human Endometrial Cancer. *Oncology* **2018**, *95*, 43–51.
150. Pan, Y.; Tong, J.H.M.; Lung, R.W.M.; Kang, W.; Kwan, J.S.H.; Chak, W.P.; Tin, K.Y.; Chung, L.Y.; Wu, F.; Ng, S.S.M.; et al. RASAL2 Promotes Tumor Progression through LATS2/YAP1 Axis of Hippo Signaling Pathway in Colorectal Cancer. *Mol. Cancer* **2018**, *17*, e102.
151. Li, N.; Li, S. RASAL2 Promotes Lung Cancer Metastasis through Epithelial-Mesenchymal Transition. *Biochem. Biophys. Res. Commun.* **2014**, *455*, 358–362.
152. Zhang, X.; Fu, L.; Xue, D.; Zhang, X.; Hao, F.; Xie, L.; He, J.; Gai, J.; Liu, Y.; Xu, H.; et al. Overexpression of Rsf-1 Correlates with Poor Survival and Promotes Invasion in Non-Small Cell Lung Cancer. *Virchows Arch.* **2017**, *470*, 553–560.
153. Kim, H.R.; Hwang, S.J.; Shin, C.H.; Choi, K.H.; Ohn, T.; Kim, H.H. SRSF3-Regulated miR-132/212 Controls Cell Migration and Invasion by Targeting YAP1. *Exp. Cell Res.* **2017**, *358*, 161–170.
154. Lai, M.; Liang, L.; Chen, J.; Qiu, N.; Ge, S.; Ji, S.; Shi, T.; Zhen, B.; Liu, M.; Ding, C.; et al. Multidimensional Proteomics Reveals a Role of UHRF2 in the Regulation of Epithelial-Mesenchymal Transition (EMT). *Mol. Cell. Proteom.* **2016**, *15*, 2263–2278.
155. Galloway, N.R.; Ball, K.F.; Stiff, T.; Wall, N.R. Yin Yang 1 (YY1): Regulation of Survivin and its Role in Invasion and Metastasis. *Crit. Rev. Oncog.* **2017**, *22*, 23–36.
156. Kuranaga, Y.; Sugito, N.; Shinohara, H.; Tsujino, T.; Taniguchi, K.; Komura, K.; Ito, Y.; Soga, T.; Akao, Y. SRSF3, a Splicer of the PKM Gene, Regulates Cell Growth and Maintenance of Cancer-Specific Energy Metabolism in Colon Cancer Cells. *Int. J. Mol. Sci.* **2018**, *19*, e3012.
157. Cancer Genome Atlas Research Network. The Molecular Taxonomy of Primary Prostate Cancer. *Cell* **2015**, *163*, 1011–1025.

© 2019 by the authors. Submitted for possible open access publication under the terms and conditions of the Creative Commons Attribution (CC BY) license (http://creativecommons.org/licenses/by/4.0/).
